# Pharmaceutical and pesticide mixtures in a Mediterranean coastal wetland: comparison of sampling methods, ecological risks, and removal by a constructed wetland

**DOI:** 10.1007/s11356-024-31968-0

**Published:** 2024-01-26

**Authors:** Claudia Martínez-Megías, Alba Arenas-Sánchez, Diana Manjarrés-López, Sandra Pérez, Yolanda Soriano, Yolanda Picó, Andreu Rico

**Affiliations:** 1 Department of Analytical Chemistry, Physical Chemistry and Chemical Engineering, University of Alcalá, Ctra. Madrid-Barcelona, Km 33.600, 28871 Alcalá de Henares, Madrid Spain; 2 IMDEA Water Institute, Parque Científico Tecnológico de La Universidad de Alcalá, Punto Com, 2, 28805 Alcalá de Henares, Madrid Spain; 3 ONHEALTH, Department of Environmental Chemistry, IDAEA-CSIC, C/Jordi Girona 18-26, 08034 Barcelona, Spain; 4 Food and Environmental Research Group of the University of Valencia (SAMA-UV), Research Desertification Centre (CIDE) (CSIC-UV-GV), CV-315 Road, Km 10.7, 46113 Moncada, Valencia Spain; 5 Cavanilles Institute of Biodiversity and Evolutionary Biology, University of Valencia, c/ Catedrático José Beltrán 2, 46980 Paterna, Valencia Spain

**Keywords:** Constructed wetlands, Contaminant mixtures, Ecological risk assessment, Environmental monitoring, Mediterranean coastal wetlands, Passive samplers

## Abstract

Pharmaceuticals and pesticides can be considered hazardous compounds for Mediterranean coastal wetland ecosystems. Although many of these compounds co-occur in environmental samples, only a few studies have been dedicated to assessing the ecotoxicological risks of complex contaminant mixtures. We evaluated the occurrence of 133 pharmaceuticals and pesticides in 12 sites in a protected Mediterranean wetland, the Albufera Natural Park (ANP), based on conventional grab sampling and polar organic chemical integrative samplers (POCIS). We assessed acute and chronic ecological risks posed by these contaminant mixtures using the multi-substance Potentially Affected Fraction (*msPAF*) approach and investigated the capacity of a constructed wetland to reduce chemical exposure and risks. This study shows that pharmaceuticals and pesticides are widespread contaminants in the ANP, with samples containing up to 75 different compounds. POCIS samplers were found to be useful for the determination of less predictable exposure profiles of pesticides occurring at the end of the rice cultivation cycle, while POCIS and grab samples provide an accurate method to determine (semi-)continuous pharmaceutical exposure. Acute risks were identified in one sample, while chronic risks were determined in most of the collected samples, with 5–25% of aquatic species being potentially affected. The compounds that contributed to the chronic risks were azoxystrobin, ibuprofen, furosemide, caffeine, and some insecticides (diazinon, imidacloprid, and acetamiprid). The evaluated constructed wetland reduced contaminant loads by 45–73% and reduced the faction of species affected from 25 to 6%. Our study highlights the need of addressing contaminant mixture effects in Mediterranean wetlands and supports the use of constructed wetlands to reduce contaminant loads and risks in areas with high anthropogenic pressure.

**Supplementary Information:**

The online version contains supplementary material available at 10.1007/s11356-024-31968-0.

## Introduction

Mediterranean coastal wetlands constitute hotspots of aquatic biodiversity and provide a large number of ecosystem services, spanning from nutrient cycling and carbon sequestration to food source provision or tourism attraction (Morant et al. 2020; Pérez-Ruzafa et al. 2011; Rodrigo et al. 2013). Despite their high ecological value, these ecosystems have been identified as being at risk due to a wide range of anthropogenic pressures (Martínez-Megías and Rico 2022). These include water scarcity related to water abstraction, climate change, increasing demographic pressure, or the expansion of agriculture. The demographic increase is one of the major causes of water contamination due to the emission of anthropogenic contaminants, including pharmaceuticals, life-style compounds, and personal care products (Sadutto et al. 2021). On the other hand, intensive agriculture has been described as one of the most detrimental activities for these ecosystems due to the emission of nutrient loads that contribute to eutrophication and the use of synthetic pesticides (Barbieri et al. 2020; Calvo et al. 2021).

Several studies performed in Mediterranean wetland ecosystems located near important urban or agricultural areas show that these aquatic ecosystems are exposed to complex contaminant mixtures, with pesticides and pharmaceuticals being among the most hazardous compounds (Barbieri et al. 2020; Sadutto et al. 2021). However, most studies evaluating their potential environmental hazard have been performed following a single substance approach (e.g., Daam et al. 2013; Gamarra et al. 2015; Sánchez et al. 2006; Shen et al. 2022). The European prospective risk assessment procedure for pesticides only considers mixture toxicity assessments when the evaluated commercial product contains more than one active ingredient (EFSA 2013), thus overlooking co-exposure with other substances applied in the same or neighboring agricultural fields (Van den Brink et al. 2018). In line with this, the evaluation of priority and preferent substances done under the umbrella of the Water Framework Directive uses environmental quality standards for a very limited number of compounds (Syberg et al. 2009), thus disregarding additive effects caused by contaminant mixtures in aquatic ecosystems.

One of the main challenges when assessing the environmental risk of contaminant mixtures relies on the complexity of capturing the right spatio-temporal dynamics of chemical exposure. Previous studies have shown that passive sampling techniques, deployed in surface waters for prolonged periods, are a good alternative to traditional grab sampling since they can incorporate temporal dynamics of chemical exposure (Hayden et al. 2022; Yabuki et al. 2018). Also, they can be seen as complementary, so while grab sampling are preferred for measuring peak concentrations of well-known chemical discharges, passive samplers offer the opportunity of capturing unpredictable exposure peaks and estimating average concentrations over prolonged time periods that are more suited for calculating chronic risks (Bernard et al. 2019). This is particularly important for pesticides, whose exposure dynamics are influenced by different application practices in heterogenous agricultural landscapes, as well as irrigation, plant wash-off, and agricultural runoff events (Yabuki et al. 2018). On the other hand, passive samplers may be seen as less relevant for down-the-drain compounds, such as pharmaceuticals, which are subject to a rather continuous emission and exposure pattern (Rico et al. 2019).

In recent years, there has been growing interest in identifying nature-based solutions to reduce pharmaceutical and pesticide loads into freshwater ecosystems. Free-water surface constructed wetlands (hereafter constructed wetlands) consist of a soil layer over which rooted vegetation can grow and that is flooded by a shallow water column. The combination of vegetation and sediment promotes the removal of contaminants by processes such as plant filtration and sediment adsorption (Stefanakis 2019) and hydrolysis or photolysis (Vymazal and Březinová 2015). Moreover, although mechanisms underlying chemical removal in constructed wetlands are interdependent, some key components that ensure their efficiency are the type of macrophytes and their uptake capacity, as well as the microbial biodegradation activity and the retention time of the running water (Stefanakis 2019). Constructed wetlands have been suggested as complementary measures to wastewater treatment facilities for nutrient sequestration to limit eutrophication (Carabal et al. 2023; Rodrigo et al. 2018). Furthermore, they can (partially) reduce the concentration of chemicals that are not easily removed by conventional wastewater treatment methods such as some pharmaceuticals or drugs of abuse (Li et al. 2014; Martín et al. 2020; Vallés et al. 2018), and can reduce agricultural pesticide loads (Rodrigo et al. 2018; Vymazal and Březinová, 2015). However, studies that assess their chemical elimination for a wide array of compounds with different physico-chemical properties and that describe their risk reduction capacity under specific Mediterranean conditions are very limited.

The aim of this study was to assess the exposure and risks of complex chemical mixtures in a protected Mediterranean wetland characterized by a high spatial heterogeneity and receiving contamination from urban and agricultural sources. Within this framework, our specific research objectives were (1) to comparatively assess the capacity of conventional grab sampling methods and passive sampling methods, based on polar organic chemical integrative samplers (POCIS), to determine pharmaceutical and pesticide exposure; (2) to assess acute and chronic ecological risks posed by chemical mixtures of these two groups of contaminants for aquatic ecosystems using a probabilistic risk assessment approach; and (3) to evaluate the capacity of constructed wetlands to reduce environmental exposure and risks for downstream freshwater ecosystems.

## Materials and methods

### Study area and sampling design

**Fig. 1:**
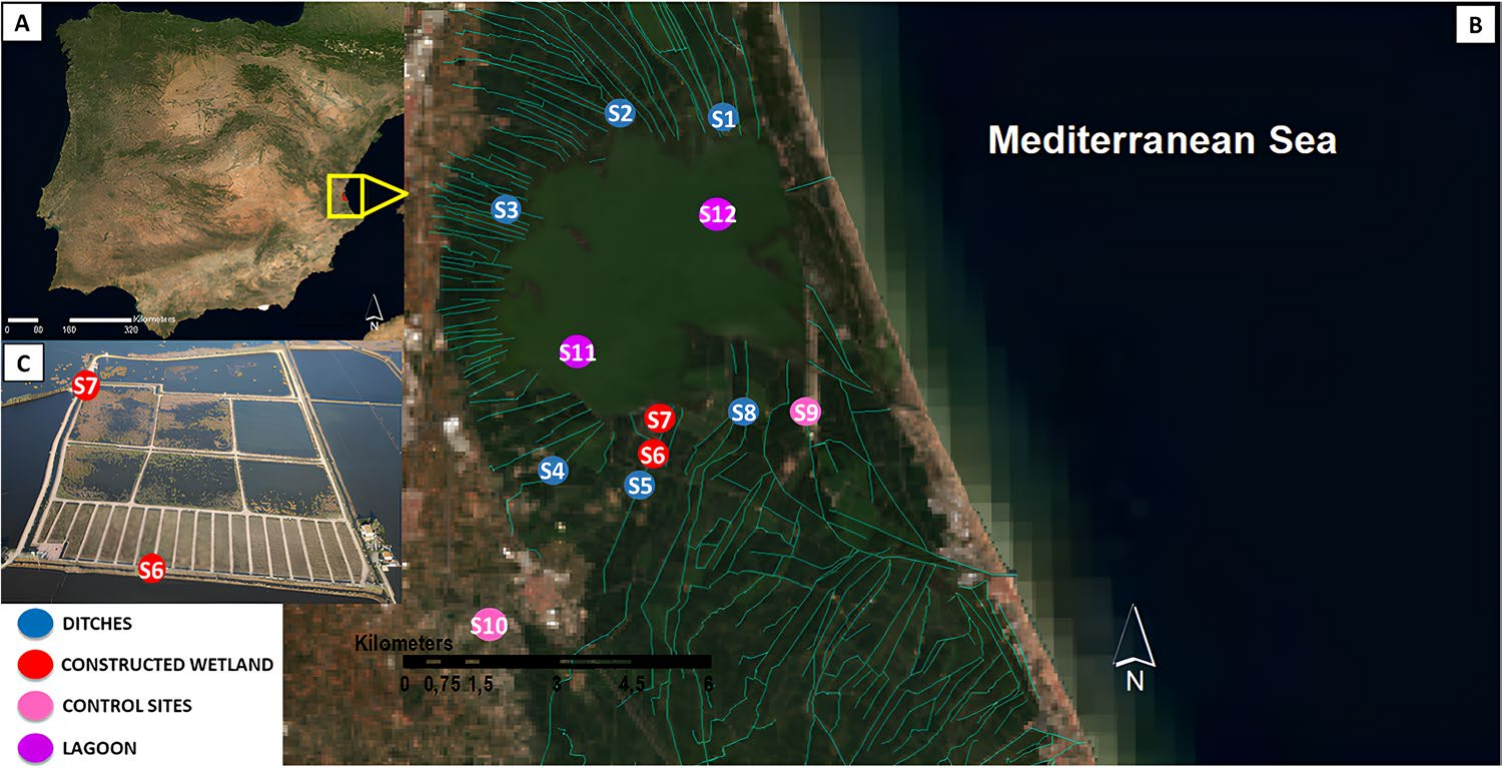
**A** Location of the Albufera Natural Park in the Iberian Peninsula. **B** Distribution of the sampling points and hydrological network in study area consisting on ditches that discharge water into the Albufera lake. **C** Constructed wetland (Tancat de Milia) with its corresponding inlet (S6) and outlet (S7) (picture provided by J. Jiménez-Romo)

This study was carried out in the Albufera Natural Park (ANP), which is located near the city of Valencia, in the Mediterranean coast of Spain (Fig. 1). This protected area comprises a coastal lagoon surrounded by marshlands and rice paddies, all interconnected by an intricate system of irrigation and drainage channels. As a result of its proximity to highly populated areas, such as the city of Valencia, the ANP receives WWTP effluents, which are used to irrigate the rice fields during the rice growing season (spring–summer).

A sampling campaign was carried out between 21 September and 8 October 2020. This period coincides with the rice harvest period and the emptying of the paddy fields, thus being a period in which pesticides are prone to be remobilized from the rice field sediments and discharged into adjacent drainage channels and the lake. Grab samples as well as POCIS samples were taken from 12 points that were established with the aim to cover a gradient of environmental conditions within the ANP (Fig. 1). These included eight points along the channels draining the rice fields, which also transported WWTP effluents into the lake (sites 1–8). Site 6 was the water inlet of the constructed wetland, which brings treated wastewater from the Albufera Sur WWTP by means of an underground pipe, and site 7 being the discharge point of the constructed wetland into the Albufera Lake. The sampling also included a pond inside a fish research center (site 9) that served as “unpolluted control” and an irrigation channel that brought water from an upstream reservoir and that was supposed to be free of pharmaceutical or pesticide residues (Sollana, site 10). Finally, two samples were taken in the south and north areas of the Albufera Lake (sites 11 and 12, respectively). The description of all the sampling points as well as their location can be found in the Supplementary Data (Table S1).

Two grab samples were taken with a 14-day time interval. The first sampling took place between 21–24 September in the different locations (hereafter D0), while the second one between 5–8 October (hereafter D14) of 2020. Grab samples were collected with poly(ethylene terephthalate) bottles (1.5 L) and were frozen at − 20 °C until further processing. Field blanks, formed by bottles of distilled water that were open in every sampling location, were used to verify that there was no contamination during the sampling or transportation. In each sampling site, physicochemical parameters were measured in situ using a multiparametric HANNA HI0194 (Supplementary Data, Table S2), and nutrient concentrations (N and P) were characterized following the methods described in APHA (2005) (Supplementary Data, Table S3).

POCIS passive samplers were purchased from USGS Technology (Columbia, MO, USA). The POCIS samplers were made of an Oasis Hydrophilic-Lipophilic Balance (HLB) sorbent, introduced into a membrane that was fixated between two stainless steel washers with a circular opening of 41 cm^2^. The structure was attached into a stainless-steel cage, which was placed on top of the sediment, allowing the POCIS sampler to be placed about 15 cm above the sediment. The POCIS samplers were deployed in the same locations where the grab samples were taken on D0, and retrieved on D14, so they were subject to a field exposure duration of 14 days. After collection, the POCIS sorbent material and membranes were introduced into air-tight amber glass bottles containing 50 mL of methanol (MeOH) analytical grade and were transported to the laboratory and stored at − 20 °C until further analysis.

The constructed wetland evaluated in this study (called “Tancat de Milia”) is an assemblage of 33 ha divided into two main areas (Fig. 1). The first one is composed of 18 sections of sub-surface constructed wetland that performs a first water treatment by promoting phytoplankton death through the absence of light, as well as solid retention by gravel and nutrient retention by the growing rhizosphere. The second one comprises six sections of surface wetlands with varying depths and vegetation cover. In these wetlands, natural retention and degradation processes facilitated by plants and microorganisms occur, allowing for further treatment of the water before discharge into the Albufera Lake (Tancats de Mília i L'Illa 2023). Some of the most representative plant species are the emergent plants *Phragmites australis*, *Typha* spp., and *Iris pseudocorus* as well as other semiaquatic plants species like *Lithrum salicaria* (Rodrigo et al. 2022). The entire wetland filtrates a total volume of 1.17 hm^3^ per year, part of which is pumped from the Albufera Lake itself (Vallés et al. 2018).

### Sample processing and chemical analysis

A total of 65 pesticides and 68 pharmaceutical compounds were analyzed (Supplementary Data, Tables S4 and S5). The selection of contaminants was based on previous literature data that have reported their occurrence in the study area (Andreu Sánchez 2008; Calvo et al. 2021; Peris et al. 2005; Sadutto et al. 2021; Vazquez-Roig et al. 2011).

#### Chemicals and reagents

The analytical standards used for the studied pharmaceuticals and pesticides were purchased from Sigma-Aldrich (Madrid, Spain). Analytical standards used as isotopically labeled standards (IS) were also purchased from Sigma-Aldrich (St. Louis, Missouri, U.SA), Toronto Research Chemicals (Toronto, Ontario, Canada), CDN Isotopes (Pointe-Claire, Quebec, Canada), and Santa Cruz Biotechnology (Dallas, TX). The IS used for pharmaceutical quantification are shown in Table S5. The IS used for the pesticides were chlorfenvinphos d10 and chlorpyrifos d10. Standards and IS were > 99% purity. Stock solutions of each target analyte were prepared in 100% acetonitrile (MeCN), 100% MeOH, 100% dimethyl sulfoxide (DMSO), or 100% HPLC water according to compound solubility. Individual IS were directly obtained as solutions at a concentration of 1 mg mL^−1^ or prepared in MeOH or DMSO at a final concentration of 1 mg mL^−1^. Working solution mixtures used for analysis and calibration purposes were prepared by serial dilution in MeOH at concentrations of 2 μg mL^−1^, whereas the IS mixture was prepared by serial dilution in MeOH at a concentration of 1 μg mL^−1^ and added to the samples as surrogate standards at 50 ng mL^−1^. All solutions were stored at − 20 °C. For the chromatographic separation and the MS analysis of the extracts, high-purity mobile phase solutions were prepared using MeCN and water (Optima™ LCMS Grade) purchased from Fisher Chemical (Fisher Scientific SL, Madrid, Spain) or ultrahigh-purity water was obtained from an Elix Milli-Q system (Millipore, Billerica, MA, USA) and MeOH (vwr International, Barcelona, Spain) with ammonium formate from Sigma-Aldrich as additive. Dichloromethane were also from VWR International. The stationary phase cartridges tested—Strata-X (33 μm, 200 mg 6 mL^−1^, polymeric reversed phase)—were from Phenomenex (Torrance, CA, USA). All glassware was cleaned by ultrasonic agitation in water containing detergent and then rinsed with ultrapure water and high-purity MeOH.

#### Sample treatment

A Solid Phase Extraction (SPE) procedure was carried out using 250 mL of the water sample, previously passed through a 0.45-μm glass fiber filter (Advantec MFS, Dublin, CA, USA). Subsequently, 50 μL of a mixture of internal standards, with a concentration of 1 μg mL^−1^, was added to the water samples. The SPE process employed Phenomenex Strata-X cartridges (33 μm, polymeric reversed phase, 200 mg 6 mL^−1^) that had been preconditioned with 6 mL of MeOH followed by 6 mL of Milli-Q water under vacuum at 400 mbar ^1^ Pa^−1^.

Once the sample loading was complete, the cartridges underwent a wash off with 6 mL of Milli-Q water and were subsequently dried for 15 min. The target analytes were eluted from the SPE cartridges using 6 mL of MeOH, followed by 3 mL of MeOH:dichloromethane (50:50 v/v). The eluates were then evaporated to dryness using a gentle stream of nitrogen at 40 °C, resulting in a residue that was redissolved in 1 mL of MeOH.

For pesticide analysis, 500 μL of the redissolved extract was filtered through a PTFE syringe filter of 13-mm diameter and 0.22 µm of pore size, and introduced into a 1.5-mL LC vial. The other 500 μL of the redissolved extract was used for pharmaceutical analysis. This extract was evaporated, dried, and then reconstituted to 500 μL using a 70:30 Milli-Q water:MeOH solution. The extract used to determine pharmaceuticals cannot be just methanol because if the chromatographic elution strength of the solvent is much higher compared to the mobile phase this may result in lower plate numbers and hence lower separation efficiency. This extract was also filtered as that for pesticides but using a filter of Nylon (other characteristics were the same), and introduced into 1.5-mL glass vials with a 250-μL polypropylene insert. The excess of the extract was discarded. Finally, both pharmaceutical and pesticide extracts were stored at − 20 °C until injection for analysis (Sadek 2002).

#### LC–MS/MS analysis

For pesticides, chromatographic separation was performed with a 1260 Infinity ultrahigh-performance LC system coupled to a 6410 triple-quadrupole mass (MS/MS) with electrospray ionization (ESI) interface from Agilent Technologies (Santa Clara, CA, USA) and according to the method described by Calatayud-Vernich et al. (2019). The mobile phase consisted of ammonium formate (10 mmol L^−1^) in MeOH (solvent A) and ammonium formate (2.5 mmol L^−1^) in water (solvent B) for positive mode. The flow rate was 0.3 mL min^−1^. The elution gradient was as follows: 0 min (50% B), 10 min (83% B), 12 min (83% B), 12.5 min (98% B), and 15.5 min (98% B). The column temperature was set at 30 °C; the injection volume was 5 μL. The analytical column was Luna C18 (15.0 cm × 0.21 cm) with a 3-μm particle size (Phenomenex, Torrance, USA). Detection was performed on the triple-quadrupole using selected reaction monitoring (SRM) using two transitions: precursor ion followed by product ion.

For pharmaceuticals, chromatographic separation was carried out with a Waters Acquity UPLC system equipped with a reversed-phase EVO C18 KINETEX column (100 × 2.1 mm, 2.6 μm). The mobile phases used for the positive electrospray ionization mode (ESI +) and (ESI −) were (A) 5 mM of ammonium acetate and 0.1% formic acid (F.A.) in H_2_O and (B) 100% MeCN, and (A) 2 mM NH_4_F in H_2_O and (B) 100% MeCN, respectively. The flow rate was 0.2 mL min^−1^; the chromatographic run was completed in 19 min. The elution gradient for both modes was as follows: 5% A (0.0 min), 30% A (10 min), 65% A (13.30 min), 100% A (15.50 min), and 5% A (17.70 min). The column temperature was set at 40 °C and the injection volume was 10 μL. Detection was performed using an Orbitrap Q-Exactive™ mass spectrometer (Thermo Fischer Scientific, San Jose, CA, USA). The acquisition was performed in full-Scan mode followed by a targeted-DIA (Data Independent Acquisition) combining the accurate mass of each compound with short RT windows according to Gómez-Navarro et al. (2023). Optimized detection parameters for the analytical method and quantification method are shown in the Supplementary Data (Tables S4 and S5). The limits of detection (LOD), limits of quantification (LOQ), and the recovery percentage for each compound are provided in Table S6. The methods used here were developed and validated according to the guidelines provided by SANTE (2020), with little adjustments for the water matrix used in this study, and are also described in Carmona et al. (2017) and Picó et al. (2021).

#### POCIS extraction and analysis

The extraction process began with the careful transfer of 50 mL of methanol (MeOH) and sorbent material from amber glass bottles to a glass funnel. This mixture was directed onto a 60-mL SPE polypropylene cartridge (Extrabond, Scharlab, Barcelona, Spain) equipped with a high-density polyethylene 20-µm frit (Agilent Technologies, Palo Alto, CA, USA). Afterward, the sorbent membrane underwent a thorough rinse with an additional 20 mL of MeOH, and the resulting solution was collected in the same flask. The extracted solution was then subjected to evaporation until dryness, effectively concentrating the analytes. The dried residue was reconstituted using 1 mL of MeOH. Then the sample followed the SPE procedure described above. Next, half of the reconstituted solution in methanol was filtered and stored for pesticides analysis, and the other half was evaporated to dryness and redissolved in 500 μL of 70:30 Milli-Q water:MeOH solution for pharmaceutical analyses. The extracts were analyzed by LC–MS/MS as described above for pesticides and pharmaceuticals.

The calculations of the mean chemical concentrations in the water samples over the 14-day exposure period with the POCIS samplers were performed according to Rico et al. (2019):

1 $${C}_{w}=\frac{{A}_{s}}{{R}_{s}t}$$

where *C*_*w*_ is the pesticide or pharmaceutical concentration in water (ng L^−1^), *A*_*s*_ is the mass of pesticide or pharmaceutical measured in the POCIS sorbent (ng), *t* is the exposure time (i.e., 14 days), and *R*_*s*_ is the sampling rate (L d^−1^) for each compound. The *R*_*s*_ values were obtained from the literature (Morin et al. 2012, 2013; Rico et al. 2019). Non-available values were approximated from the octanol–water partition coefficients (*K*_*ow*_) of the chemical compounds. For pesticides, the *R*_*s*_ values were set to 0.2 or 0.3 when their log *K*_*ow*_ values were between 0.5 and 3 or 3 and 5, respectively. For pesticides with log *K*_*ow*_ values outside this range and for pharmaceuticals, the *R*_*s*_ values were estimated according to Rico et al. (2019):

2 $$R_s=0.08+0.02\;\log\;K_{ow}$$

The concentrations obtained from the grab samples and the POCIS samplers were compared by a Spearman rank correlation test for each group of compounds (i.e., fungicides, herbicides, insecticides, and pharmaceuticals).

### Ecological risk assessment

The ecological risk assessment of the pharmaceutical and pesticide mixtures contained in the grab and the POCIS samples was performed following a probabilistic risk assessment approach following Rico et al. (2021). First, the *µ* (median) and *σ* (slope) of the acute and chronic Species Sensitivity Distributions (SSD) for each of the evaluated substances were obtained from Posthuma et al. (2019). These SSDs had been built assuming a log-normal distribution with EC50 (Effect Concentration for 50% of individuals) for acute toxicity data and NOECs (No Observed Effect Concentrations) for chronic toxicity data including a wide range of relevant aquatic species groups (i.e., bacteria, algae, invertebrates, and fish).

Then the fraction of species affected by the mixture of compounds belonging to the same Toxic Mode of Action (TMoA), the so-called multi-substance potentially affected fraction (*msPAF*), was calculated assuming concentration addition:

3 $${msPAF}_{TMoA,i}=\int \nolimits_{-\infty}^{\log(\sum_{i=1}^n{HU}_{TMoA,i})}\frac1{\sigma_{TMoA}^2\sqrt{2\pi}}\text{exp}\frac{-\text{log}{(\sum_{i=1}^n{HU}_{TMoA,i})}^2}{2\sigma_{TMoA}^2}d\;\log\;(\sum\nolimits_{i=1}^n{HU}_{TMoA,i})$$

where the *msPAF*_*TMoA,i*_ is the multi-substance potentially affected fraction for every toxic mode of action, the *σ*_*TMoA*_ is the average standard deviation for the compounds within the same TMoA, and *HU*_*TMoA,i*_ is the calculated hazard unit for each compound belonging to the same toxic mode of action. The classification of each compound into a specific TMoA was initially performed based on the chemical groups shown in Table 1 but was later modified according to the methods described in Rico et al. (2021).

**Table 1:** Pesticide and pharmaceutical concentrations quantified in the samples taken with the grab and POCIS sampling methods in the study area

Compound	Chemical group	Water				POCIS			
		Min	Max	Mean	Freq. (%)	Min	Max	Mean	Freq. (%)
Fungicides									
Carbendazim	Benzimidazole	0.2	13	3	100	0.15	5	1	100
Tebuconazole	Benzimidazole	2	128	50	75	0.64	445	92	100
Thiabendazole	Benzimidazole	2	24	6	92	0.11	37	6	100
Imazalil	Imidazole	2	15	5	25	2.56	77	14	83
Prochloraz	Imidazole	1	21	6	63	0.77	40	20	92
Azoxystrobin	Strobilurin	2	6500	786	100	-	-	-	-
Herbicides									
Propanil	Anilide	3	24	14	13	7.72	52	32	50
Metolachlor	Chloroacetanilide	-	-	-	-	0.70	30	8	100
Diuron	Phenylamine	5	32	10	29	2.70	131	31	67
Isoproturon	Phenylurea	-	-	-	-	0.11	1	0.4	42
Atrazine	Triazine	1	2	1	50	0.31	2	1	100
*Atrazine-desethyl*	Atrazine metabolite	1	197	14	100	0.70	62	11	75
*Atrazine-desisopropyl*	Atrazine/simazine metabolite	2	6	4	25	7.69	51	34	100
Simazine	Triazine	1	5	2	63	1.03	8	4	75
Terbumeton	Triazine	0.4	3	1	63	0.08	1	0.5	100
*Terbumeton desethyl*	Terbumeton metabolite	0.5	68	20	83	0.19	30	8	100
Terbuthylazine	Triazine	0.4	3	1	75	0.12	2	1	100
*Terbuthylazine-2OH*	Terbuthylazine metabolite	1	18	6	83	0.44	6	2	100
*Terbuthylazine-desethyl*	Terbuthylazine metabolite	1	51	15	83	0.28	15	4	100
Terbutryn	Triazine	0.4	7	2	63	0.18	7	2	92
Insecticides									
*DMPF*	Amidine	15	42	25	13	2.27	9	6	67
Hexythiazox	Carboxamide/thiazolidine	-	-	-	-	0.05	0.4	0.2	42
Fipronil	Fipronil	0.5	5	1	71	0.17	1	0.4	100
*Carbofuran-3-OH*	Carbofuran metabolite	-	-	-	-	0.40	1	1	17
Spinosad A	Microorganism derived	1	1	1	4	0.04	0.4	0.2	42
Spinosad D	Microorganism derived	1	1	1	4	0.05	0.2	0.1	25
Acetamiprid	Neonicotinoid	1	84	15	100	0.37	12	4	100
Imidacloprid	Neonicotinoid	1	52	8	100	0.21	8	2	75
Chlorpyriphos	Organophosphate	-	-	-	-	1.94	78	20	92
Diazinon	Organophosphate	1	8	5	8	0.21	1	1	50
Dimethoate	Organophosphate	2	18	8	13	3.12	4	3	17
Ethion	Organophosphate	-	-	-	-	0.08	0.1	0.1	8
Fenthion sulfoxide	Organophosphate	0.5	9	4	17	0.36	2	1	17
Acrinathrin_aduct	Pyrethroid	-	-	-	-	0.32	0.3	0.3	8
Fluvalinate	Pyrethroid	-	-	-	-	0.13	0.3	0.2	42
Buprofezine	Unclassified IGR	-	-	-	-	0.08	0.1	0.1	8
Pharmaceuticals									
Acetaminophen	Analgesic	1	50	8	96	1	9	4	33
Codeine	Analgesic	1	11	3	42	0.3	1	1	25
Diclofenac	Analgesic	1	57	8	92	0.2	28	10	50
*4-Hydroxydiclofenac*	Diclofenac metabolite	1	165	112	21	-	-	-	-
Flufenamic_acid	Analgesic	7	24	16	21	1	10	6	25
Propyphenazone	Analgesic and anti-pyretic	0.3	1	1	29	0.1	0.4	0.3	33
Methadone^*^	Analgesic anesthetics	0.2	9	3	25	0.0	1	0.5	25
Tramadol	Analgesic/anesthetics	1	427	67	100	0.3	77	17	100
Ciprofloxacin^*^	Antibacterial	1	5	2	21	0.2	0.2	0.2	25
Clarithromycin^*^	Antibacterial	10	21	14	21	0.4	1	0.5	17
Triclocarban	Antibacterial	1	1	1	4	-	-	-	-
Trimethoprim	Antibacterial	1	4	1	21	1	1	1	17
1H-benzotriazole	Antibacterial, anti-fungal	0.4	22312	1652	96	3	1155	237	100
*5-methyl-1H_Benzotriazole*	Benzotriazole metabolite	1	758	129	100	2	337	78	100
Chloramphenicol	Antibiotic	0.3	1	1	100	0.1	0.1	0.1	8
Erythromycin	Antibiotic	1	7	2	33	0.3	1	0.5	17
Sulfamethazine	Antibiotic	1	11	6	8	1.1	1	1	8
Nalidixin_acid	Antibiotics/antibacterial	1	1	1	33	0.1	0.1	0.1	25
Acridone	Anti-cancer activity	2	2	2	4	3	8	4	25
Warfarin	Anti-coagulant	1	1	1	79	-	-	-	-
O-desmethylvenlafaxine	Anti-depressant	1	236	43	96	0.2	25	7	92
Temazepam	Anti-depressant, sedative, hypnotic, anti-convulsant	2	53	12	83	0.1	5	2	83
Carbamazepine	Anti-epileptic	0.2	31	8	100	0.1	9	2	100
*CBZ-10,11-epoxide*	Carbamazepine metabolite	1	22	6	54	0.3	3	2	25
Lamotrigine	Anti-epileptic	1	142	36	100	1	50	15	100
Fluconazole	Anti-fungal	1	37	15	67	0.2	13	5	83
Atenolol	Anti-hypertensive	0.2	13	3	58	6	9	7	25
Losartan_Potassium	Anti-hypertensive	0	115	29	46	0.1	9	4	50
Sulfamethoxazole	Anti-infective and antibacterial	1	66	11	79	0.4	52	14	58
*N-acethyl_SMX*	Sulfamethoxazole metabolite	1	6	2	25	0.0	0.04	0.04	33
Ibuprofen	Anti-inflammatory	30	1229	170	100	0.3	27	9	67
Indomethacin	Anti-inflammatory	0.4	0.37	0.37	8	-	-	-	-
Ketoprofen	Anti-inflammatory/anti-rheumatic	3	40	17	21	1	29	8	58
Quetiapine	Anti-psychotic agent	-	-	-	-	0.1	1	0.3	25
Alprazolam	Anxiolytic	1	1	1	33	0.1	1	0.5	42
Lorazepam	Anxiolytic	3	26	10	42	0.3	5	2	50
Sotalol	B-blocking agent	1	7	4	25	0.4	1	1	25
Diltiazem^*^	Calcium channel blocker	25	28	27	8	-	-	-	-
Verapamil	Calcium channel blocking agent	-	-	-	-	0.1	0.07	0.07	8
Metoprolol	Cardiovascular system	1	2	1	21	0.0	0.3	0.1	17
Valsartan	Cardiovascular system	3	409	72	92	0.3	63	15	100
Valsartan acid	Cardiovascular system	1	434	104	96	-	-	-	-
Caffeine	CNS stimulant	2	555	46	100	6	66	19	100
Cocaine	CNS stimulant	0.4	4	2	21	-	-	-	-
*Benzoylecgonine*	Cocaine metabolite	0.3	90	7	96	0.2	14	2	83
*Coca-ethylene*	Cocaine metabolite	1	1	1	4	-	-	-	-
Furosemide	Diuretic	1	35	7	58	2	8	5	17
Hydrochlorothiazide	Diuretic	0.2	484	49	100	0.4	240	44	100
Metformin^*^	Drug used in diabetes	0.2	8	3	25	0.1	13	3	42
Sitagliptin	Drug used in diabetes	1	296	52	67	0.2	76	20	67
Atorvastatin	Lipid regulator	1	7	2	54	-	-	-	-
Bezafibrate	Lipid regulator	58	5331	400	100	0.1	0.1	0.1	17
*Oseltamivir-CBX*	Oseltamivir metabolite	1	1	1	4	-	-	-	-
Mefenamic acid	Non-steroidal anti-inflammatory drug (NSAID)	1	1	1	4	0.1	0.3	0.1	42
Salicylic acid	Non-steroidal anti-inflammatory drug (NSAID)	8	47	21	100	4	19	10	100
Amantadine	Parkinson’s treatment	1	40	10	58	0.3	2	1	50
Omeprazole^*^	Proton-pump inhibitor	0.2	0.20	0.20	8	8	8	8	8
Nicotine^*^	Psychostimulant	0.2	367	35	88	0.03	311	37	83
*Cotinine*	Nicotine metabolite	5	123	23	100	0.04	4	1	100
Citalopram^*^	Psychoanaleptic	1	21	6	29	0.1	6	2	33
N-desmethylcitalopram^*^	Citalopram metabolite	1	23	9	21	0.4	2	1	17
Fluoxetine	Psychoanaleptic	4	5	5	8	-	-	-	-
Venlafaxine	Psychoanalytic	0.4	140	16	100	0.1	26	5	100
Diazepam	Psycholeptic	1	17	4	67	4	19	11	17
Oxazepam	Psycholeptic and anxiolytic	1	44	13	54	0.1	8	3	75
Salbutamol	Short-acting β2 adrenergic receptor agonist	1	3	1	21	2	8	4	58
Zolpidem	Treatment of insomnia	0.2	0.20	0.20	8	0.02	0.02	0.02	8
Sulfapyridine	Veterinary pharmaceutical	2	53	14	33	1	15	3	100

Concentrations are expressed in ng L^−1^. *Min*: minimum; *Max*: maximum; *Freq*: frequency of samples in which the compound was detected; *n.d.*: not detected. Metabolites are indicated in italics. The complete table showing the concentration of each compound in each sample is provided in the Supplementary Data (Table S8). ^*^The compounds marked were outside Quality Control (QC) range (i.e. the measured concentrations may be less reliable)

The hazard unit for a compound *i* is calculated as follows:

4 $${HU}_{i}=\frac{{MEC}_{i}}{{10}^{{\mu }_{i}}}$$

where *HU*_*i*_ is the hazard unit for each compound, the *MEC*_*i*_ is its measured environmental concentration in a sample, and the *µ*_*i*_ the median of its SSD calculated as the mean of the log-transformed EC50 or NOEC values for the acute and chronic SSDs, respectively. The acute risk was calculated with the highest MEC of the two sampling dates per site (D0 and D14), while the chronic risk was calculated with the mean MEC of the two sampling dates.

Finally, the total toxicity of the sample (*msPAF*_*Total*_) was calculated assuming response addition between different TMoAs contained in the same sample, according to

5 $${msPAF}_{Total}=1-{\prod }_{i=1}^{n}\left(1-{msPAF}_{TMoA,i}\right)$$

The relative contribution of each chemical to the total toxicity of the mixture represented by the the *msPAF*_*Total*_ of each sample was calculated. High acute or chronic ecological risks were assumed when the individual PAF of each substance (calculated with Eq. (3) for a single compound) or the calculated *msPAF*_*Total*_ was higher than 5%, meaning that more than 5% of the species in the ecosystem may be affected by the compound or the compound mixture contained in the sample, respectively.

## Results and discussion

### Pharmaceutical and pesticide exposure

A total of 94 compounds were detected in the grab samples, with 30 compounds being detected in more than 80% of the samples (Table 1). Azoxystrobin, carbendazim, and thiabendazole were the fungicides that were most frequently detected; the metabolites atrazine-desethyl, terbumeton desethyl, terbuthylazine-2OH, and terbuthylazine-desethyl were the most frequently detected herbicides; and acetamiprid and imidacloprid the most frequently detected insecticides. Finally, pharmaceuticals were the most frequently detected group of substances, constituting 73% of the total detected compounds. Among them, the most frequently detected compounds were distributed mainly within the anti-inflammatory, anti-depressant, and anti-hypertensive groups. Moreover, there were 21 pharmaceuticals with a frequency of detection above 80%. Among them, it is worthy to mention the lipid regulator bezafibrate, the anti-fungal 5-methyl-1H-benzotriazole, the analgesics tramadol and ibuprofen, and the anti-hypertensive hydrochlorothiazide, which occurred in 100% of the samples. The fungicide azoxystrobin and the pharmaceuticals ibuprofen, bezafibrate, and 1H-benzotriazole were the compounds showing the highest concentration per sample, being over 1000 ng L^−1^. In addition, the fungicide tebuconazole, atrazine-desethyl, and other 14 pharmaceuticals showed concentrations over 100 ng L^−1^.

The compounds detected in higher frequency (i.e., more than 80%) in the POCIS samples were the fungicides carbedanzim, tebuconazole, thiabendazole, imazalil, and prochloraz; the herbicides metolachlor, atrazine, terbumeton, terbuthylazine, and terbutryn; and the insecticides fipronil, acetamiprid, and chlorpyriphos. Regarding pharmaceuticals, there were 17 compounds with high frequencies of detection like tramadol, the derivatives of benzotriazole, the anti-hypertensive hydrochlorothiazide, and the anti-epileptic lamotrigine. The compound showing the highest calculated concentration in the POCIS samples was 1H-benzotriazole (concentration higher than 1000 ng L^−1^), while tebuconazole, diuron, and the pharmaceuticals hydrochlorothiazide, 5-methyl-1H-benzotriazole, and nicotine showed mean water concentrations over 100 ng L^−1^ (Table 1).

**Fig. 2:**
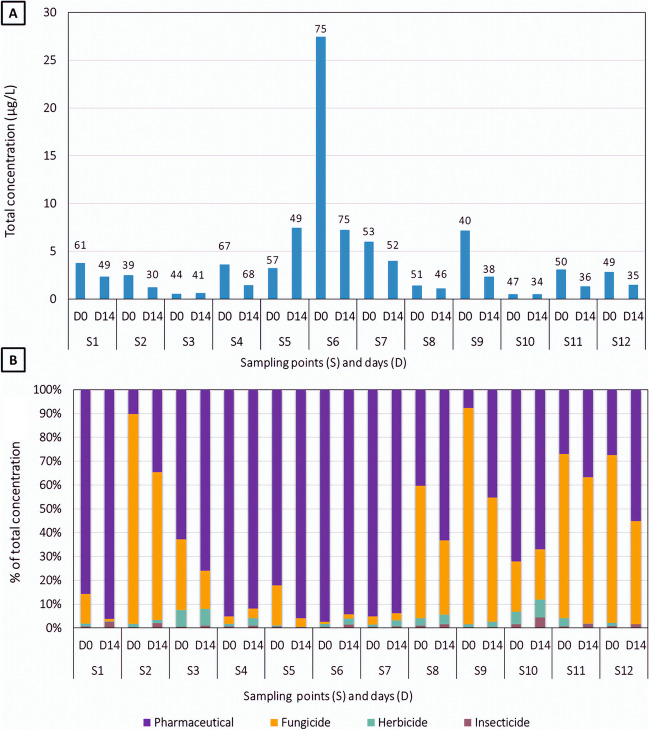
Total exposure concentration (**A**) and relative contribution of the different chemical groups to the total exposure concentration (**B**) in the grab samples. In A, the numbers above the bars represent the number of compounds detected in each sample

Site S6 (entry of the constructed wetland) was the sampling site with the highest number of detected compounds, with a total of 75 compounds in both sampling dates (Fig. 2A). Following this, the ditch channels S1 and S5 presented up to 61 and 57 compounds in D0, respectively, and S4 up to 68 compounds in D14. Surprisingly, site S9 corresponding to the fish research center and established as one of the control points in the ANP showed 40 compounds at D0. Regarding concentrations, site S6 showed the highest total concentration (27 µg L^−1^) in D0, followed by S5, S7, and S9 with total concentrations about 7 µg L^−1^ in at least one of the two grab samples. In these sites, the total concentration was dominated by pharmaceuticals. Insecticides were the most outstanding group in sites S2 and S9, both at D0 and D14, representing also more than 80% of the total concentration (Fig. 2B). On the other hand, when comparing the results of the D0 and D14 grab samplings, we could observe an overall decrease in the number of identified compounds and in the total concentration in D14 as compared to D0 (except for S4 and S5). Such a decrease of total concentrations with time could be related to the stages of the rice crop. At D0 farmers were emptying their fields for rice harvesting, which can carry residues of pesticides or pharmaceuticals accumulated in the rice paddy, while on D14 most of them had emptied them already and the water flow in the ditches was much lower.

There are several studies that reported similar pharmaceutical contamination patterns in this study area. For example, Sadutto et al. (2021) reported caffeine in 100% of grab samples at concentrations up to 555 ng L^−1^, half the concentrations registered here. Similarly to our study, they found acetaminophen in 96% of the samples at concentrations up to 168 ng L^−1^. They also detected ibuprofen in 30% of the samples but at slightly lower concentrations compared to our findings (217 vs. 4090 ng L^−1^). Conversely, Vazquez-Roig et al. (2011) also identified ibuprofen and acetaminophen at concentrations of 290 ng L^−1^and 1204 ng L^−1^, respectively, but with detection frequencies below 66%, while in our study the concentrations barely reached 180 ng L^−1^. These differences could be explained due to the spatial variability between their sampling points, located near to the effluent discharge point of WWTPs, and the ones in our study, which had a larger agricultural influence.

Regarding pesticides, Calvo et al. (2021) reported fungicides as the most frequently detected type of pesticides in water samples of the ANP, while herbicides were mostly found as degradation products. However, they recorded much greater concentrations of all grouped pesticides in some habitats (up to 10 µg L^−1^ in ditches), probably because they carried out the sampling campaign during the whole rice cultivation period, while our samples were taken at the end of the growing season, about 2 months later than the last herbicide application (Martínez-Megías et al. 2023). In the Ebro River Basin, an area that is also dominated by intensive rice farming, Ccanccapa et al. (2016) detected several compounds that have been banned for use in agriculture in Europe: the insecticides chlorpyriphos and diazinon (in more than 90% of the samples) and the fungicide carbendazim (in 70% of the samples). The current illegal status of these compounds could explain that chlorpyriphos was not detected in our study and diazinon was only found in 8% of samples; however, carbendazim was detected in all samples (at trace levels), together with azoxystrobin. Finally, the monitoring study conducted by Barbieri et al. (2020), also in the Ebro Delta, found the herbicide propanil in 83% of samples at concentrations up to 61 × 10^3^ ng L^−1^, while in our study it was detected only at 13% of samples and with maximum concentrations of 24 ng L^−1^. This could be related to the sampling period, which was closer to the herbicide application moment in the study carried out by Barbieri et al. (2020). The same situation occurred with other compounds typically applied in rice paddies like acetamiprid that was detected at concentrations up to 4000 ng L^−1^, in contrast with the maximum of 84 ng L^−1^ recorded here.

### Comparison between POCIS and grab sampling

**Fig. 3:**
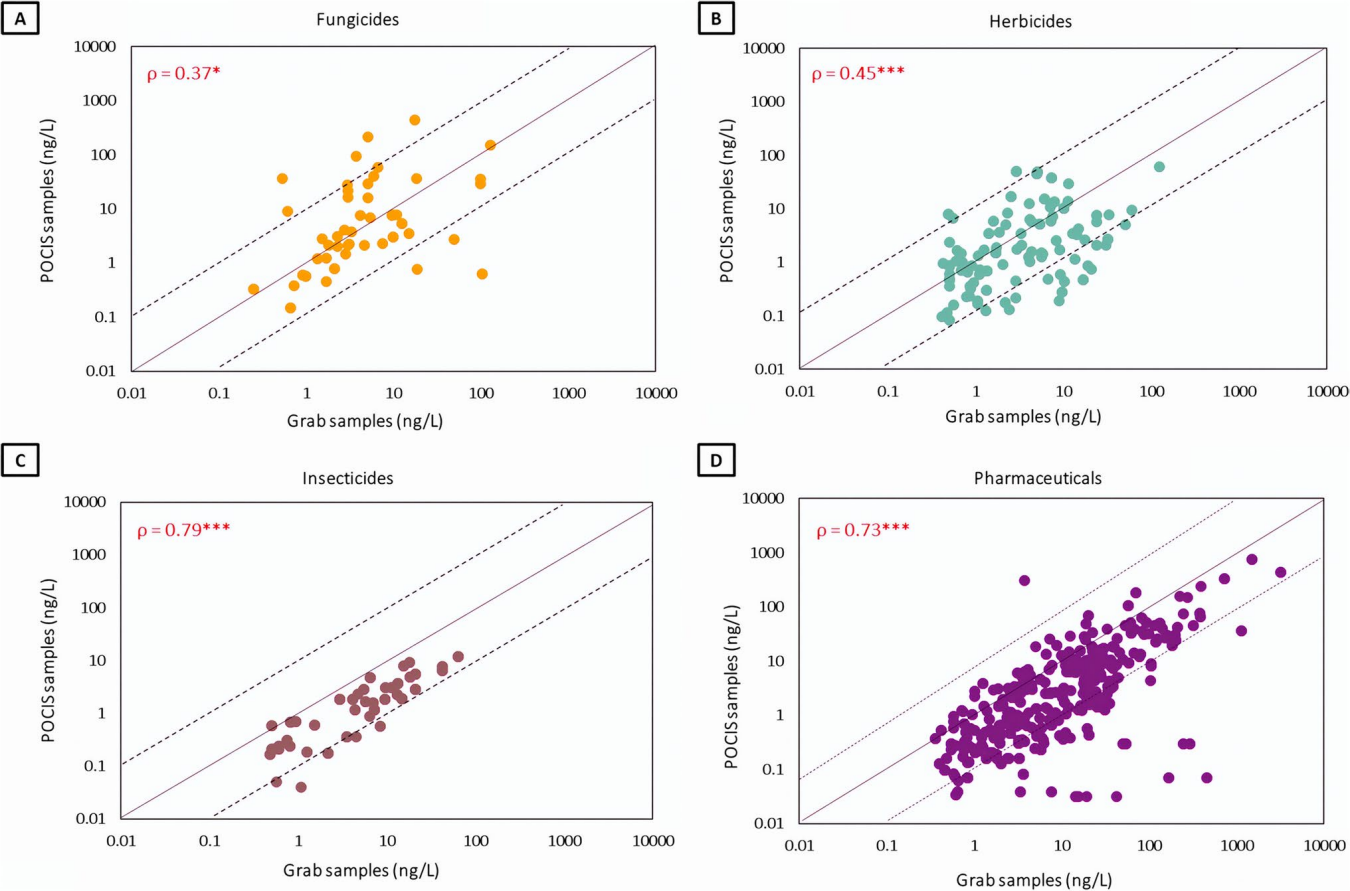
Comparison of chemical concentrations measured in the grab and POCIS samples. Observations outside the dot line interval represent deviations above 1 order of magnitude between both sampling methods. *ρ* is the Spearman’s rank correlation coefficient between the concentrations obtained with the grab and the POCIS sampling methods. The asterisks indicate a *p*-value of ≤ 0.05 (*); ≤ 0.01 (**); and ≤ 0.001 (***)

The comparison of the chemical exposure concentrations obtained with the grab samples (mean of the D0 and D14 samples) and the POCIS samplers is shown in Fig. 3. The results of the Spearman rank tests indicated a significant correlation between both methods for all contaminant groups (*p*-value < 0.05). However, the correlation for insecticides and pharmaceuticals was stronger (Spearman’s rank correlation coefficient, *ρ* > 0.7) than for herbicides and fungicides (Spearman’s *ρ* = 0.3–0.5). The number of cases in which the differences between both sampling methods exceeded or fell below 1 order of magnitude was 33% for fungicides, 47% for herbicides, 58% for insecticides, and 53% for pharmaceuticals (Supplementary Data, Table S7). For fungicides, more than 90% of these deviations corresponded to imazalil, prochloraz, and tebuconazole. These compounds have a log *K*_*ow*_ higher than 3 (Chemspider 2023a, b, c), which means that they are less prone to be captured by POCIS (Alvarez et al. 2007). However, in most cases these fungicides were detected at higher concentrations by POCIS, except one sample of prochloraz in S7 and two samples of tebuconazole on S4 and S10 that were detected at higher concentrations in grab samples. Regarding herbicides, those that showed a larger deviation between both sampling methods were metolachlor and the metabolite atrazine-desisopropyl. Metolachlor was only detected in POCIS, while atrazine-desisopropyl was found at higher concentrations in the POCIS samples than in the grab samples. The insecticides chlorpyriphos, fluvalinate, and hexythiazox were only detected in the POCIS samplers. Finally, some pharmaceuticals such as atorvastatin, bezafibrate, chloramphenicol, valsartan acid, and warfarin were not detected by the POCIS samplers, and compounds such as ibuprofen, temazepam, and cotinine (metabolite of nicotine) were consistently found at higher concentrations in the grab samples in all sampling points.

The concentrations of the grab samples taken on D14 showed a better correlation to the results of the POCIS samples as compared to those measured on D0, especially for fungicides and herbicides (Supplementary Data Figure S1). It is expected that concentration peaks were more common on D0 due to emptying of the rice fields. For example, imazalil was better detected by the POCIS because probably the emissions of this fungicide during the emptying of the rice fields were less constant over time and the grab sampling did not capture well the concentration peaks. On the other hand, the correlations between the grab samples and the POCIS samples for insecticides and pharmaceuticals were rather similar between D0 and D14. The prevention to insect pests that affects rice crops and other agricultural orchards in the surroundings of the ANP usually occurs in spring or early summer, so the residual concentrations measured here rather represent residues of applications that occurred about 2 months prior to the sampling moment.

Regarding the spatial variability, the results showed that S4 (Alqueresía channel) was the site with the best matching between the grab and the POCIS samples, with 62% of chemical comparisons having a ratio between 0.1 and 10, followed by S2 (Comú channel) and S1 (Tancaeta channel), with 59% and 56%, respectively. Also, S4 and S2 presented a relatively low concentration of pollutants, in contrast to S6 which was the most polluted site. On the other hand, S5 (Els Campets channel) was the site with the worst matching between both sampling methods, followed by S10 (Sollana channel) and S3 (Font Nova channel). These last two points were the sites showing the lowest pollution levels, both in terms of total concentration and number of detected compounds. In S3, the compounds with ratio lower than 0.1 (i.e., higher concentrations in POCIS) were 21% and the ones better measured by grab samples were 37%. On the other hand, the S10 site showed that 44% of compounds had a ratio over 10 (i.e., higher concentrations in grab samples), suggesting the possibility that peak concentrations did take place in this site. In the sites with ratios exceeding 10 (S5, S10, and S3), pharmaceuticals were the predominant group. Conversely, compounds with ratios below 0.1 were primarily dominated by pesticides, particularly herbicides. The S2, S3, and S10 sites were also partially governed by fungicide exposure, with azoxystrobin and tebuconazole showing the highest concentrations. Therefore, a clear spatial correlation cannot be inferred from these results.

According to Guibal et al. (2018), both grab and POCIS sampling are complementary techniques for pesticide monitoring, since they observed that grab sampling allowed to detect peak concentrations of some compounds, while POCIS permitted to identify a higher number of substances even at low concentrations. However, the results obtained by Rico et al. (2019) in the Tagus River basin (Spain), which have similar climate conditions to ANP, demonstrated a higher sensitivity of POCIS than grab sampling in detecting and quantifying pesticides, as observed in our study for some fungicides. The combination of both grab sampling and POCIS seems important for environments with constant but also peak exposure patterns (Bernard et al. 2019). This fact is supported by the monitoring guidelines established by the Water Framework Directive, which strongly encourages the addition of passive sampling techniques for areas subjected to river flow variations or discontinuous chemical emissions (Criquet et al. 2017), such as our study area. Unlike pesticides, the traditional grab sampling still seems to be an effective tool to the monitoring of certain substances like pharmaceuticals since their exposure profile is usually more constant than for pesticides. Moreover, it was found that up to 44% of the POCIS samples did not accurately measure these compounds.

Discrepancies between grab and POCIS samples may arise from multiple factors. The most important have been discussed above and relate to the differences in the exposure profile of the evaluated compounds, which can induce to larger or lower concentrations by grab sampling than POCIS depending on the moment that the sample is taken. Another important factor is the calibration of POCIS samplers for different compounds and environmental scenarios. The calibration of POCIS analytical extractions and sampling rates is a challenging task that has been investigated by several studies (see review by Wang et al. 2020). Studies show that laboratory calibrations may not fully capture the diverse range of results observed under different environmental scenarios (Zhang et al. 2008), mainly because the *R*_*s*_ of different compound groups varies according to a number of environmental factors. The most important factor is the flow rate, while temperature, pH, and the ionization potential of the compound, dissolved organic matter, or the biofilm layers formed over the POCIS membrane also play a significant role (Wang et al. 2020). The influence of each of these parameters in every environmental scenario is difficult to predict, and this also applies to our sampling sites, which were subject to different water dynamics (e.g., drainage channels vs lake) and degrees of eutrophication. The contaminant nature also plays an important role in the *R*_*s*_. Some studies have demonstrated a positive correlation between the *R*_*s*_ and the log *K*_*ow*_ of pharmaceutical and personal care products (Li et al. 2010; Rico et al. 2019), which usually reaches a plateau when a log *K*_*ow*_ of 4 is exceeded (Ibrahim et al. 2013). Therefore, we should acknowledge that some uncertainty in our POCIS calculations may reflect the variability between the *R*_*s*_ values taken from the literature, or the extrapolated ones based on the log *K*_*ow*_, and the actual ones corresponding to our sampling sites. Further implementations of this technique would require in-situ calibration exercises with a selected number of compounds (e.g., Zhang et al. 2008), as performing such exercise with the extensive list of compounds investigated here would be unaffordable given to the number of samples and compounds to be analyzed under each environmental scenario. Our study helps to indicate compounds that show maximal discrepancies between the grab and POCIS sampling and that should be considered the primary target of such calibration exercises under Mediterranean wetland conditions.

### Ecological risk assessment

**Fig. 4:**
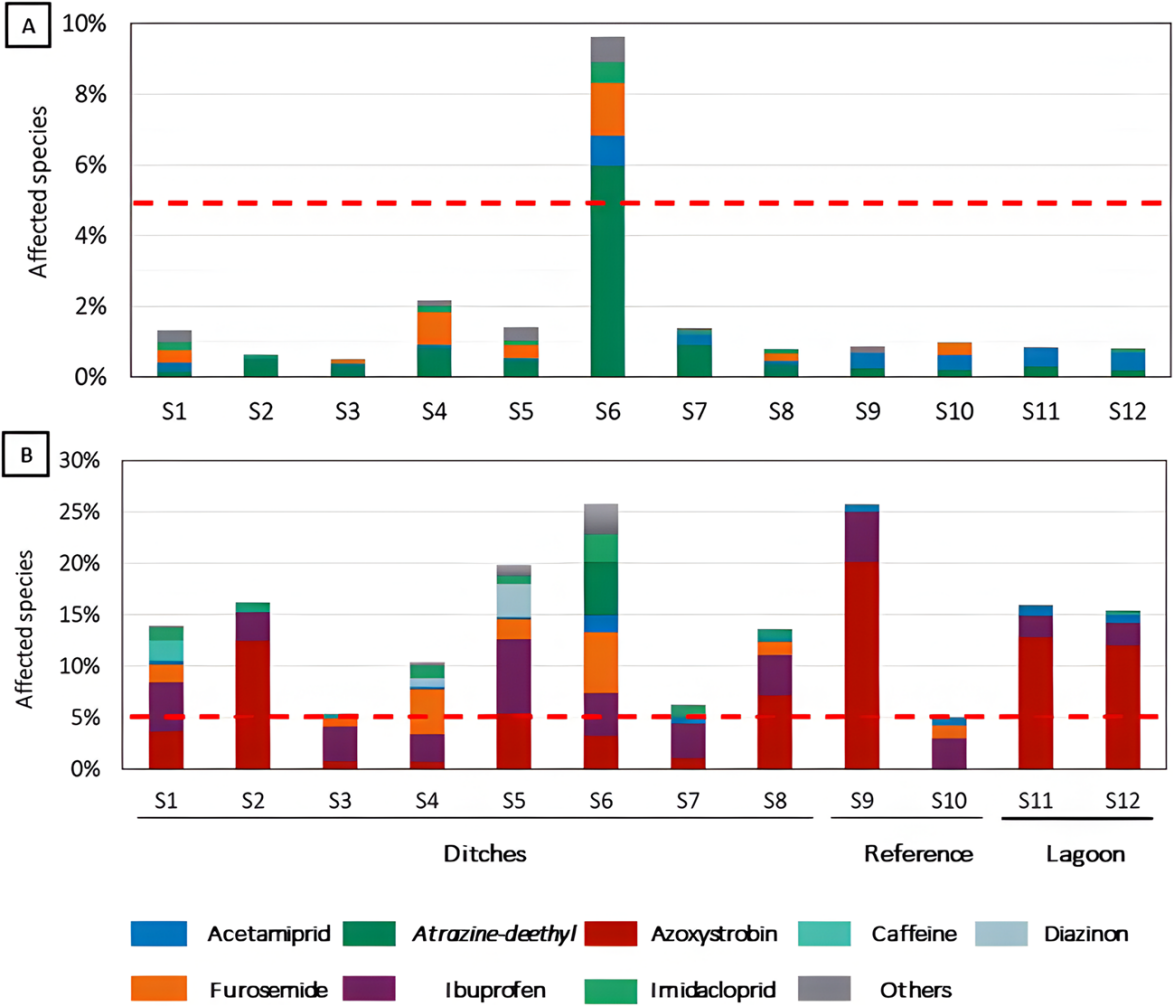
Calculated acute (**A**) and chronic (**B**) *msPAF*_*Total*_ in each sampling site. Red striped line represents a threshold above which high ecological risks are expected (5% of species). The colors indicate the relative contribution of each compound to the calculated *msPAF*_*Total*_ in each sampling site. Only compounds with a calculated individual PAF above 1% are displayed, while the rest are grouped as “Others.” The acute and chronic PAFs for each compound in each sample are provided in the Supplementary Data (Table S9)

The results of the *msPAF*_*Total*_ calculations for grab sampling (Fig. 4) showed notable differences between the acute and the chronic risk assessment. Regarding acute risks (Fig. 4A), the highest *msPAF*_*Total*_ value was calculated in the constructed wetland entry (S6), with more than 9% of species potentially affected, mainly due to the herbicide metabolite atrazine-desethyl. The rest of sampling sites had *msPAF*_*Total*_ acute values below 5% of species, which suggest insignificant risks. Regarding chronic risks, we found that the threshold of the 5% of species was exceeded in all sites. Sites S6 and S9 showed the highest chronic risks, with *msPAF*_*Total*_ values above 20% of species, followed by sites S1, S2, S4, S5, S11, and S12 with *msPAF*_*Total*_ values 10–20% and S3, S7, and S10 with *msPAF*_*Total*_ values between 5 and 10%. The compound that contributed most to the chronic risks was azoxystrobin, followed by ibuprofen; the diuretic furosemide; caffeine; the insecticides diazinon, imidacloprid, and acetamiprid; and the metabolite atrazine-desethyl (affecting more than 5% of species in at least one sample; Fig. 4B). Previous studies have identified caffeine and tramadol as the two most important substances affecting algae, invertebrates, and fish in the study area (Sadutto et al. 2021). In our study, caffeine was also identified as one of the most hazardous compounds. Calvo et al. (2021) pointed at several of these substances as highly hazardous for aquatic organisms, including the herbicides propanil, the insecticide acetamiprid, and the fungicides prochloraz and tebuconazole, all of them closely related to rice cultivation practices and consistent with the results obtained here.

It is important to note that previous risk assessment studies in the study area have been based on PNECs derived with standard test species and the application of assessment factors, which may overestimate risks for some taxonomic groups. At the same time, such methods usually do not take into account risks caused by complex contaminant mixtures. Here we found that risks were driven by a combination of 2–3 compounds for the acute assessment and a combination of 2–6 substances for the chronic risk assessment. This is in line with other studies that found that ecological risks are usually driven by a reduced number of compounds at the local scale (De Zwart and Posthuma 2005; Molnar et al. 2021). However, our study shows that performing a risk assessment using a single compound approach would underestimate risks up to 4% and 20% for the acute and chronic risk assessments, respectively, supporting the need of taking complex contaminant mixtures into account. This study also shows that many compounds contributing to the ecological risk are hardly monitored as part of the regular monitoring programs in the study area and should therefore be included as priority substances in further monitoring and ecotoxicological studies.

### Effects of the constructed wetland on reducing contaminant risks

**Fig. 5:**
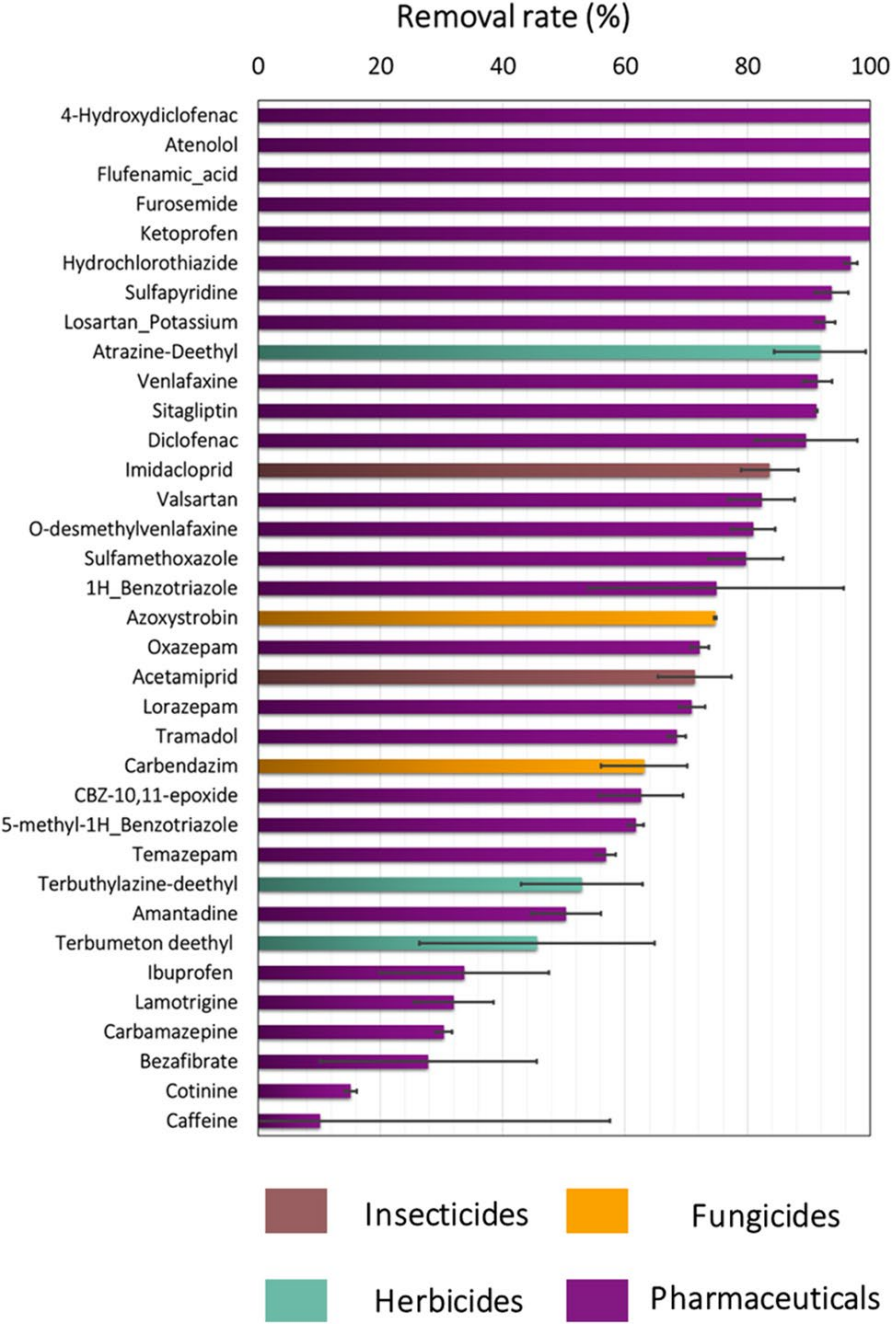
Mean percentage of elimination of chemicals by the constructed wetland. Only compounds whose measured concentration at the entry of the constructed wetland was higher than 10 ng L^−1^ are displayed. Error bars represent the standard error of the mean

The contaminant removal by the constructed wetland was expressed as the percentage of concentration decrease in the outlet (S7) as compared to the concentration in the inlet (S6) considering both sampling dates (Fig. 5). Overall, the constructed wetland lowered the total chemical concentration from 27.5 to 6 µg L^−1^ on D0, and 7.3 to 4 µg L^−1^ on D14, with a total removal rate of 73% and 45% when considering all compounds on D0 and D14, respectively. Insecticides were the contaminant group with the highest mean removal efficiency (80%), followed by pharmaceuticals and herbicides (70%), and fungicides (10%; Supplementary Data Figure S2). The latter was largely influenced by the contamination with tebuconazole, which showed a higher concentration in the outlet than in the inlet, probably due to spray-drift deposition from aerial applications in the surrounding rice fields. The calculated removal rate for the different compounds is shown in Fig. 5. About 50% of the evaluated compounds showed a removal rate of 80% or higher, including the herbicide metabolite atrazine-desethyl, the insecticide imidacloprid, and 17 pharmaceuticals (see Fig. 5). The contaminant removal resulted in a notable ecological risk reduction at the outlet of the constructed wetland (Fig. 3), with acute *msPAF* values being reduced from 7 to about 1%, and chronic *msPAF* values from 25 to 6%. The compounds that were significantly removed and that contributed most to lower the ecotoxicological risk at the outlet of the constructed wetland were atrazine-desethyl, furosemide, imidacloprid, and azoxystrobin.

Together with the study by Rodrigo et al. (2022) our study is one of the few studies that explored the removal efficiency of contaminants of emerging concern in constructed wetlands of the Mediterranean region. The study by Rodrigo et al. (2022) found 65 pesticides inside the constructed wetland and 29 outside, and calculated removal rates of 50% for fungicides, 64% for herbicides, and 47% for insecticides, which are similar to the mean percentages found in our study. Pharmaceutical compounds such as ibuprofen, acetaminophen, diclofenac, sulfamethoxazole, or diazepam usually show low removal efficiencies after the implementation of conventional methods in WWTPs, and some authors have pointed to the need to develop alternative methods for them (Couto et al. 2019; Vona et al. 2015). The current study shows that the evaluated constructed wetland can be regarded as an efficient method to reduce exposure concentrations of the anti-inflammatory drug diclofenac or the antibiotic sulfamethoxazole (among others), with removal rates above 80%, although it seems to be less promising for the analgesic ibuprofen or the anti-epileptics lamotrigine and carbamazepine which did not reach removal rates of 50% (Fig. 5). In general terms, the elimination efficacy can be related to the polarity of the molecules and consequently their log *K*_*ow*_ (Matamoros and Rodríguez 2016; Vymazal and Březinová 2015), although sulfamethoxazole (log *K*_*ow*_ < 3) and ibuprofen (log *K*_*ow*_ > 3) represent an exception.

## Conclusions

Our study shows that pharmaceuticals and pesticides are widespread contaminants in the Albufera Lake and the surrounding ditches, with some samples containing between 50 and 75 different compounds. The comparison of grab and POCIS sampling techniques shows that the latter can be more effective to detect peak exposure concentrations that occur during hours or days in the water bodies, such as those created by pesticides when emptying the rice fields. On the other hand, grab sampling is a robust method to assess pharmaceutical exposure. Our study shows that ecological risks were driven by mixtures of 2–7 compounds, so that the application of the single-compound approach commonly used by the regulatory assessment performed in these ecosystems may underestimate ecological risks. The mixture assessment method employed here shows that high chronic risks may be expected in the majority of the sampling sites, with the fungicide azoxystrobin, ibuprofen, furosemide, caffeine, and some insecticides (diazinon, imidacloprid, and acetamiprid) being the main responsible for those. It should be noted, however, that these results become relevant for the period of the year evaluated in this study, and other compounds and mixtures, including other metabolites not included in this study, may drive the risk in other periods of the rice cultivation period. Finally, our study shows that the evaluated constructed wetland (‘Tancat de Milia’) significantly reduces contaminant loads into the protected Albufera Lake and can be considered as an efficient nature-based solution to lower the ecological risk of pharmaceutical and pesticide pollution in Mediterranean coastal wetlands.

### Supplementary Information

Below is the link to the electronic supplementary material.

Supplementary file1 (DOCX 2301 KB)

Supplementary file2 (XLSX 101 KB)

## Data Availability

Further data and details corresponding to this study are available upon request to the corresponding author.

## References

[CR1] Alvarez DA, Huckins JN, Petty JD, Jones-Lepp T, Stuer-Lauridsen F, Getting DT, Goddard JP, Gravell A (2007) Tool for monitoring hydrophilic contaminants in water: polar organic chemical integrative sampler (POCIS). In Greenwood R, Mills G, Vrana B (Eds.), Comprehensive analytical chemistry 1st ed., Vol. 48, pp 171–197

[CR2] Andreu Sánchez OE (2008) Evaluación de riesgos ambientales del uso de plaguicidas empleados en el cultivo del arroz en el Parque Natural de La Albufera de Valencia. Doctoral dissertation, Universitat Politècnica de València. Available at: https://riunet.upv.es/bitstream/id/5446/tesisUPV2815.pdf

[CR3] APHA, American Publich Health Association (2005) Standard methods for the examination of water and wastewater. American Water Works Association/American Public Works Association/Water Environment Federation. 10.2105/AJPH.51.6.940-a

[CR4] Barbieri MV, Peris A, Postigo C, Moya-Garcés A, Monllor-Alcaraz LS, Rambla-Alegre M, Eljarrat E, de López Alda M (2020) Evaluation of the occurrence and fate of pesticides in a typical Mediterranean delta ecosystem (Ebro River Delta) and risk assessment for aquatic organisms. Environ Pollut 274. 10.1016/j.envpol.2020.115813 10.1016/j.envpol.2020.115813 33257154

[CR5] Bernard M, Boutry S, Lissalde S, Guibaud G, Saüt M, Rebillard J, Mazzella N (2019). Combination of passive and grab sampling strategies improves the assessment of pesticide occurrence and contamination levels in a large-scale watershed. Sci Total Environ.

[CR6] Calatayud-Vernich P, Calatayud F, Simó E, Pascual Aguilar JA, Picó Y (2019). A two-year monitoring of pesticide hazard inhive: high honey bee mortality rates during insecticide poisoning episodes in apiaries located near agricultural settings. Chemosphere.

[CR7] Calvo S, Romo S, Soria J, Picó Y (2021). Pesticide contamination in water and sediment of the aquatic systems of The Natural Park of the Albufera of Valencia (Spain) during the rice cultivation period. Sci Total Environ.

[CR8] Carabal N, Segura M, Puche E, Rojo C, Rodrigo MA (2023). How the diversity of constructed wetlands improves the plankton communities discharged into a protected Mediterranean wetland. Hydrobiologia.

[CR9] Carmona E, Andreu V, Picó Y (2017). Multi-residue determination of 47 organic compounds in water, soil, sediment and fish—Turia River as case study. J Pharm Biomed Anal.

[CR10] Ccanccapa A, Masiá A, Navarro-Ortega A, Picó Y, Barceló D (2016). Pesticides in the Ebro River basin: occurrence and risk assessment. Environ Pollut.

[CR11] Chemspider (2023a) CSID:34116. http://www.chemspider.com/Chemical-Structure.34116.html. Accessed 23 Aug 2023

[CR12] Chemspider (2023b) CSID:66316. http://www.chemspider.com/Chemical-Structure.66316.html. Accessed 23 Aug 2023

[CR13] Chemspider (2023c) CSID:77680. http://www.chemspider.com/Chemical-Structure.77680.html. Accessed 23 Aug 2023

[CR14] Couto CF, Lange LC, Amaral MC (2019). Occurrence, fate and removal of pharmaceutically active compounds (PhACs) in water and wastewater treatment plants—a review. J Water Process Eng.

[CR15] Criquet J, Dumoulin D, Howsam M, Mondamert L, Goossens JF, Prygiel J, Billon G (2017). Comparison of POCIS passive samplers vs. composite water sampling: a case study. Sci Total Environ.

[CR16] Daam MA, Santos Pereira AC, Silva E, Caetano L, Cerejeira MJ (2013). Preliminary aquatic risk assessment of imidacloprid after application in an experimental rice plot. Ecotoxicol Environ Saf.

[CR17] De Zwart D, Posthuma L (2005). Hazard/risk assessment complex mixture toxicity for single and multiple species: proposed methodologies. Environ Toxicol Chem.

[CR18] European Parliament & Council (2000) Directive 2000/60/EC of 23 October 2000 establishing a framework for Community action in the field of water policy. Official Journal of the European Communities L327:1–72

[CR19] EFSA (2013) Guidance on tiered risk assessment for plant protection products for aquatic organisms in edge-of-field surface waters. EFSA J 11(7). 10.2903/j.efsa.2013.3290

[CR20] Gamarra JS, Godoi AFL, de Vasconcelos EC, de Souza KMT, Ribas de Oliveira CM (2015). Environmental Risk Assessment (ERA) of diclofenac and ibuprofen: a public health perspective. Chemosphere.

[CR21] Gómez-Navarro O, Labad F, Manjarres-López DP, Pérez S, Montemurro N (2023) HRMS-targeted-DIA methodology for quantification of wastewater-borne pollutants in surface water. MethodsX 10(102093). 10.1016/j.chroma.2021.462760 10.1016/j.mex.2023.102093 PMC10011426 36926270

[CR22] Guibal R, Lissalde S, Leblanc J, Cleries K, Charriau A, Poulier G, Mazzella N, Rebillard JP, Brizard Y, Guibaud G (2018). Two sampling strategies for an overview of pesticide contamination in an agriculture-extensive headwater stream. Environ Sci Pollut Res.

[CR23] Hayden KR, Preisendanz HE, Elkin KR, Saleh LB, Weikel J, Veith TL, Elliott HA, Watson JE (2022). Comparison of POCIS and grab sampling techniques for monitoring PPCPs in vernal pools in central Pennsylvania. Sci Total Environ.

[CR24] Ibrahim I, Togola A, Gonzalez C (2013). Polar organic chemical integrative sampler (POCIS) uptake rates for 17 polar pesticides and degradation products: laboratory calibration. Environ Sci Pollut Res.

[CR25] Li H, Helm PA, Metcalfe CD (2010). Sampling in the Great Lakes for pharmaceuticals, personal care products, and endocrine-disrupting substances using the passive polar organic chemical integrative sampler. Environ Toxicol Chem.

[CR26] Li Y, Zhu G, Ng WJ, Tan SK (2014). A review on removing pharmaceutical contaminants from wastewater by constructed wetlands: design, performance and mechanism. Sci Total Environ.

[CR27] Martín M, Hernández-Crespo C, Andrés-Doménech I, Benedito-Durá V (2020). Fifty years of eutrophication in the Albufera lake (Valencia, Spain): causes, evolution and remediation strategies. Ecol Eng.

[CR28] Martínez-Megías C, Mentzel S, Fuentes-Edfuf Y, Moe SJ, Rico A (2023). Influence of climate change and pesticide use practices on the ecological risks of pesticides in a protected Mediterranean wetland: a Bayesian network approach. Sci Total Environ.

[CR29] Martínez-Megías C, Rico A (2022) Biodiversity impacts by multiple anthropogenic stressors in Mediterranean coastal wetlands. Sci Total Environ 818. 10.1016/j.scitotenv.2021.151712 10.1016/j.scitotenv.2021.151712 34800444

[CR30] Matamoros V, Rodríguez Y (2016). Batch vs continuous-feeding operational mode for the removal of pesticides from agricultural run-off by microalgae systems: a laboratory scale study. J Hazard Mater.

[CR31] Molnar E, Fodor I, Svigruha R, Pirger Z (2021) Issues, challenges, directives, and limitations concerning the improvement of environmental risk assessment of pharmaceutically active compounds. Ecotoxicol Environ Saf 216. 10.1016/j.ecoenv.2021.112212 10.1016/j.ecoenv.2021.112212 33839486

[CR32] Morant D, Picazo A, Rochera C, Santamans AC, Miralles-Lorenzo J, Camacho-Santamans A, Ibañez C, Martínez-Eixarch M, Camacho A (2020). Carbon metabolic rates and GHG emissions in different wetland types of the Ebro Delta. PLoS One.

[CR33] Morin N, Miège C, Coquery M, Randon J (2012). Chemical calibration, performance, validation and applications of the polar organic chemical integrative sampler (POCIS) in aquatic environments. TrAC - Trends Analyt Chem.

[CR34] Morin N, Camilleri J, Cren-Olivé C, Coquery M, Miège C (2013). Determination of uptake kinetics and sampling rates for 56 organic micropollutants using “pharmaceutical” POCIS. Talanta.

[CR35] Pérez-Ruzafa A, Marcos C, Pérez-Ruzafa IM (2011). Mediterranean coastal lagoons in an ecosystem and aquatic resources management context. Phys Chem Earth.

[CR36] Peris E, Requena S, De La Guardia M, Pastor A, Carrasco JM (2005). Organochlorinated pesticides in sediments from the Lake Albufera of Valencia (Spain). Chemosphere.

[CR37] Picó Y, Campo J, Alfarhan AH, El-Sheikh MA, Barceló D (2021) A reconnaissance study of pharmaceuticals, pesticides, perfluoroalkyl substances and organophosphorus flame retardants in the aquatic environment, wild plants and vegetables of two Saudi Arabia urban areas: environmental and human health risk assessment. Sci Total Environ 776. 10.1016/j.scitotenv.2021.145843 10.1016/j.scitotenv.2021.145843 33640550

[CR38] Posthuma L, de Zwart D, Dyer SD (2019). Chemical mixtures affect freshwater species assemblages: from problems to solutions. Curr Opin Environ Sci Health.

[CR39] Rico A, Arenas-Sánchez A, Alonso-Alonso C, López-Heras I, Nozal L, Rivas-Tabares D, Vighi M (2019). Identification of contaminants of concern in the upper Tagus river basin (central Spain). Part 1: screening, quantitative analysis and comparison of sampling methods. Sci Total Environ.

[CR40] Rico A, de Oliveira R, de Souza Nunes GS, Rizzi C, Villa S, López-Heras I, Vighi M, Waichman AV (2021) Pharmaceuticals and other urban contaminants threaten Amazonian freshwater ecosystems. Environ Int 155. 10.1016/j.envint.2021.106702 10.1016/j.envint.2021.106702 34139589

[CR41] Rodrigo MA, Martín M, Rojo C, Gargallo S, Segura M, Oliver N (2013). The role of eutrophication reduction of two small man-made Mediterranean lagoons in the context of a broader remediation system: effects on water quality and plankton contribution. Ecol Eng.

[CR42] Rodrigo MA, Valentín A, Claros J, Moreno L, Segura M, Lassalle M, Vera P (2018). Assessing the effect of emergent vegetation in a surface-flow constructed wetland on eutrophication reversion and biodiversity enhancement. Ecol Eng.

[CR43] Rodrigo MA, Puche E, Carabal N, Armenta S, Esteve-Turrillas FA, Jiménez J, Juan F (2022). Two constructed wetlands within a Mediterranean natural park immersed in an agrolandscape reduce most heavy metal water concentrations and dampen the majority of pesticide presence. Environ Sci Pollut Res.

[CR44] Sadek PC (2002). Solvent miscibility and viscosity chart. The HPLC solvent guide.

[CR45] Sadutto D, Andreu V, Ilo T, Akkanen J, Picó Y (2021). Pharmaceuticals and personal care products in a Mediterranean coastal wetland: impact of anthropogenic and spatial factors and environmental risk assessment. Environ Pollut.

[CR46] Sánchez P, Kubitza J, Peter Dohmen G, Tarazona JV (2006). Aquatic risk assessment of the new rice herbicide profoxydim. Environ Pollut.

[CR47] SANTE (2020) SANTE/2020/12830, Rev.1 24. February 2021. Guidance document on pesticide analytical methods for risk assessment and post-approval control and monitoring purposes. Available at: https://food.ec.europa.eu/system/files/2021-03/pesticides_ppp_app-proc_guide_res_mrl-guidelines-2020-12830.pdf. Accessed 10 May 2023

[CR48] Shen C, Pan X, Wu X, Xu J, Dong F, Zheng Y (2022). Ecological risk assessment for difenoconazole in aquatic ecosystems using a web-based interspecies correlation estimation (ICE)-species sensitivity distribution (SSD) model. Chemosphere.

[CR49] Stefanakis AI (2019). The role of constructed wetlands as green infrastructure for sustainable urban water management. Sustainability (switzerland).

[CR50] Syberg K, Jensen TS, Cedergreen N, Rank J (2009). On the use of mixture toxicity assessment in reach and the water framework directive: a review. Hum Ecol Risk Assess.

[CR51] Tancats de Mília i L'Illa. Humedales artificiales en l'Albufera (2023). Inicio. Los Tancats de Milia Y L'Illa. Funcionamiento. Available at:Albufera.bio/index.php/es. Accessed on 14^th^ June 2023.

[CR52] Vallés FJ, Martín M, Nácher B, Hernández-Crespo C, Andrés-Doménech I, Eguibar MA, Gargallo S, Albentosa E (2018). Manual técnico para una gestion optima de la hidráulica en humedales restaurados para mejora del habitat y de la calidad del agua.

[CR53] Van den Brink PJ, Boxall ABA, Maltby L, Brooks BW, Rudd MA, Backhaus T, Spurgeon D, Verougstraete V, Ajao C, Ankley GT, Apitz SE, Arnold K, Brodin T, Cañedo-Argüelles M, Chapman J, Corrales J, Coutellec MA, Fernandes TF, Fick J, … van Wensem J (2018) Toward sustainable environmental quality: priority research questions for Europe. Environ Toxicol Chem 37(9):2281–2295. 10.1002/etc.4205 10.1002/etc.4205 PMC6214210 30027629

[CR54] Vazquez-Roig P, Andreu V, Onghena M, Blasco C, Picó Y (2011). Assessment of the occurrence and distribution of pharmaceuticals in a Mediterranean wetland (L’Albufera, Valencia, Spain) by LC-MS/MS. Anal Bioanal Chem.

[CR55] Vona A, di Martino F, Garcia-Ivars J, Picó Y, Mendoza-Roca JA, Iborra-Clar MI (2015). Comparison of different removal techniques for selected pharmaceuticals. J Water Process Eng.

[CR56] Vymazal J, Březinová T (2015). The use of constructed wetlands for removal of pesticides from agricultural runoff and drainage: a review. Environ Int.

[CR57] Wang L, Liu R, Liu X, Gao H (2020). Sampling rate of polar organic chemical integrative sampler (POCIS): influence factors and calibration methods. Appl Sci.

[CR58] Yabuki Y, Ono J, Nagai T, Inao K, Tanimori S (2018). Determining the suitability of a polar organic chemical integrated sampler ( POCIS ) for the detection of pesticide residue in the Ishikawa River and its tributary in Osaka, Japan. J Pestic Sci.

[CR59] Zhang Z, Hibberd A, Zhou JL (2008). Analysis of emerging contaminants in sewage effluent and river water: comparison between spot and passive sampling. Anal Chim Acta.

