# Supplementary material for: Pharmaceutical and pesticide mixtures in a Mediterranean coastal wetland: comparison of sampling methods, ecological risks, and removal by a constructed wetland
Source: Environ Sci Pollut Res Int. 2024 Jan 26;31(10):14593–609. doi: 10.1007/s11356-024-31968-0 (PMC10884053; doi:10.1007/s11356-024-31968-0)

**Supplementary Data 1**

**Complex pharmaceutical and pesticide mixtures in a protected Mediterranean wetland: environmental occurrence, comparison of sampling methods and ecological risk assessment**

Authors: Claudia Martínez-Megías^1,2^, Alba Arenas-Sánchez^2^, Diana Manjarrés-López^3^, Sandra Pérez^3^, Yolanda Soriano^4^, Yolanda Picó^4^, Andreu Rico^2,5*^

^1^University of Alcalá, Department of Analytical Chemistry, Physical Chemistry and Chemical Engineering, Ctra. Madrid-Barcelona KM 33.600, 28871, Alcalá de Henares, Madrid, Spain

^2^IMDEA Water Institute, Punto Com, 2, Parque Científico Tecnológico de la Universidad de Alcalá 28805, Alcalá de Henares, Madrid, Spain

^3^ONHEALTH, Department of Environmental Chemistry, IDAEA-CSIC, c/Jordi Girona 18-26, 08034, Barcelona, Spain

^4^Food and Environmental Research Group of the University of Valencia (SAMA-UV), Research Desertification Centre (CIDE) (CSIC-UV-GV), Moncada-Naquera Road, Km 4.5, 46113 Moncada, Valencia, Spain

^5^Cavanilles Institute of Biodiversity and Evolutionary Biology, University of Valencia, c/ Catedrático José Beltrán 2, 46980, Paterna, Valencia, Spain

*Corresponding author: Andreu Rico; email: [andreu.rico@uv.es](mailto:andreu.rico@uv.es)

**Table S1**. GPS coordinates and characteristics of the different sampling sites within the Albufera Natural Park.

| **Site code** | **Name** | **GPS coordinates** | **Characteristics** |
| --- | --- | --- | --- |
| S1 | Tancaeta (channels Fus or Ravisancho) | 39.362013, -0.339671 | Irrigation/drainage channel-Rice fields |
| S2 | Comú channel | 39.3649860, -0.3617570 | Irrigation/drainage channel-Rice fields |
| S3 | Font Nova channel | 39.3464890, -0.3870840 | Irrigation/drainage channel-Rice fields |
| S4 | Alqueresía/Rizarbe channel | 39.3008557, -0.3759367 | Irrigation/drainage channel-Rice fields |
| S5 | Els Campets channel | 39.305112, -0.356743 | Irrigation/drainage channel-Rice fields |
| S6 | Tancat de Milia, constructed wetland (inlet) | 39.305392, -0.356989 | Treated WWTP effluent |
| S7 | Tancat de Milia, constructed wetland (outlet) | 39.311023,  -0.356076 | WWTP effluent after constructed wetland |
| S8 | Obera channel | 39.3096979, -0.3365428 | Irrigation/drainage channel-Rice fields |
| S9 | C.I.P El Palmar | 39.310954,  -0.321044 | Control point: research centre |
| S10 | Sollana channel | 39.2379140, -0.4112020 | Control point: Irrigation channel |
| S11 | Albufera (south) | 39.320680, -0.368986 | Lake (south point) |
| S12 | Albufera (north) | 39.344191, -0.358216 | Lake (north point) |

**Table S2.** Physicochemical parameters measured in the sampling sites (mean±SD). DO: dissolved oxygen; Ch-a: chlorophyll-a; PE: phycoerythrin.

| **Site code** | **pH** | **Temperature (°C)** | **Conductivity (µS/cm)** | **Salinity (PSU)** | **DO (%)** | **DO (ppm)** | **Chl-a**  **(µg L^-1^)** | **PE**  **(µg L^-1^)** |
| --- | --- | --- | --- | --- | --- | --- | --- | --- |
| S1 | 7.58 ± 0.05 | 20.6±2.42 | 1865±36.23 | 0.95±0.07 | 58.6±19.14 | 5.2±1.44 | 19.7±7.11 | 41.2±16.4 |
| S2 | 7.63 ± 0.07 | 20.1±2.21 | 1781±59.73 | 0.91±0.03 | 70.5±8.44 | 6.4±0.89 | 10.4±3.03 | 20.8±5.61 |
| S3 | 7.58 ± 1.77 | 20.8±1.77 | 1698±16.41 | 0.86±0.01 | 64.4±15.77 | 5.8±1.57 | 4.7±0.32 | 7.8±0.92 |
| S4 | 7.58± 0.1 | 21.0±1.9 | 1393±50 | 0.70±0.03 | 89.4±8.7 | 8.0±0.9 | 0.96±0.56 | 2.3±1.3 |
| S5 | 7.60± 0.1 | 19.7±1.8 | 1589±140 | 0.80±0.1 | 68.0±7.8 | 6.2±0.9 | 3.0±1.57 | 6.3±3.6 |
| S6 | 7.43± 0.1 | 23.2±1.3 | 2060±70 | 1.05±0.04 | 20.4±4.2 | 1.7±0.4 | 0.94±0.13 | 1.7±0.3 |
| S7 | 8.14± 0.1 | 22.5±3 | 3064±50 | 1.60±0.03 | 119±26.1 | 10.4±2.1 | 17.0±14.49 | 40.1±22.3 |
| S8 | 7.61± 0.1 | 20.0±1.7 | 1465±20 | 0.74±0.01 | 69.5±6.7 | 6.3±0.8 | 2.35±1.33 | 5.01±2.7 |
| S9 | 7.97± 0.1 | 19.1±2.8 | 1519±60 | 0.77±0.03 | 87.3±1.2 | 8.1±1.2 | 2.30±0.27 | 4.63±0.5 |
| S10 | 8.22 ± 0.13 | 22.1±2.17 | 1067±7.59 | 0.53±0 | 114.8±3.68 | 10.1±0.33 | 0.11±0.06 | 0.56±0.18 |
| S11 | 8.32 ± 0.15 | 20.6±2.14 | 1840±90.88 | 0.95±0.03 | 126.9±8.75 | 11.4±0.98 | 13.9±3.2 | 34.4±9.6 |
| S12 | 8.38 ± 0.1 | 20.5±2.15 | 2031±36.23 | 1.04±0.02 | 122.8±6.01 | 10.9±1.16 | 17.9±9.34 | 42.5±23.33 |

**Table S3.** Measured nutrient concentrations in the different sampling sites. The ´Day´ column refers to the day in which the samples were taken (D0, D14).

| **Site code** | **Day** | **NO_3_^-^**  **(mg/L)** | **NO_2_^-^**  **(mg/L)** | **NH_4_^+^**  **(mg/L)** | **Total N**  **(mg/L)** | **Total P**  **(µg/L)** | **PO_4_^-3^**  **(µg/L)** |
| --- | --- | --- | --- | --- | --- | --- | --- |
| S1 | D0 | 13.0 | 0.496 | 0.597 | 3.54 | 108.2 | 66.6 |
| S2 | D0 | 31.8 | 0.187 | 0.134 | 7.35 | 63.2 | 73.0 |
| S3 | D0 | 57.2 | 0.482 | 0.254 | 13.3 | 93.2 | 95.2 |
| S4 | D0 | 35.5 | 0.275 | 0.251 | 8.28 | 42.6 | 58.8 |
| S5 | D0 | 54.3 | 0.225 | 0.451 | 12.7 | 70.8 | 75.8 |
| S6 | D0 | 16.1 | 0.012 | 0.060 | 3.69 | 40.1 | 73.5 |
| S7 | D0 | 1.18 | 0.004 | 0.142 | 0.378 | 55.1 | 119.1 |
| S8 | D0 | 28.8 | 0.146 | 0.082 | 6.60 | 43.6 | 45.1 |
| S9 | D0 | 0.458 | 0.004 | 0.144 | 0.217 | 96.4 | 66.1 |
| S10 | D0 | 4.27 | 0.010 | 0.056 | 1.01 | 26.8 | 44.2 |
| S11 | D0 | 2.94 | 0.060 | 0.155 | 0.803 | 27.3 | 7.3 |
| S12 | D0 | 1.27 | 0.026 | 0.149 | 0.410 | 73.9 | 36.5 |
| S1 | D14 | 27.4 | 1.41 | 1.54 | 7.80 | 83.3 | 81.3 |
| S2 | D14 | 64.5 | 0.384 | 0.166 | 14.8 | 73.3 | 91.8 |
| S3 | D14 | 70.9 | 0.238 | 0.192 | 16.2 | 75.8 | 100.1 |
| S4 | D14 | 32.1 | 0.172 | 0.226 | 7.49 | 44.4 | 57.1 |
| S5 | D14 | 49.5 | 0.097 | <0,05 | 11.2 | 46.8 | 72.3 |
| S6 | D14 | 16.4 | 0.055 | 0.061 | 3.77 | 266.4 | 78.3 |
| S7 | D14 | 1.11 | 0.007 | <0,05 | 0.252 | 67.6 | 81.8 |
| S8 | D14 | 53.6 | 0.175 | 0.189 | 12.3 | 57.4 | 69.8 |
| S9 | D14 | 0.786 | 0.031 | <0,05 | 0.187 | 31.1 | 40.0 |
| S10 | D14 | 3.55 | 0.011 | <0,05 | 0.804 | 47.1 | 50.8 |
| S11 | D14 | 0.975 | 0.005 | <0,05 | 0.222 | 51.4 | 60.1 |
| S12 | D14 | 4.38 | 0.067 | 0.119 | 1.10 | 44.4 | 55.9 |

**Table S4.** MRM conditions used in the HPLC-MS/MS determination of pesticides.

| **Target Pesticide** | **RT^(a)^**  **(min)** | **Precursor Ion** | **Production (MS1)** | **Fragmentor (V)** | **CE ^(b)^ (V)** | **Product ion (MS2)** | **Fragmentor (V)** | **CE^(b)^ (V)** |
| --- | --- | --- | --- | --- | --- | --- | --- | --- |
| Acetamiprid | 2.3 | 223 | 126 | 111 | 22 | 56 | 111 | 14 |
| Acetochlor | 13.1 | 270 | 224 | 120 | 10 | 148 | 120 | 10 |
| Acrinathrin | 18.4 | 559 | 208 | 76 | 10 | 181 | 76 | 30 |
| Alachlor | 13.09 | 270 | 238 | 80 | 10 | 162 | 80 | 15 |
| Atrazine | 9.06 | 216 | 174 | 120 | 15 | 132 | 120 | 20 |
| Atrazine-desethyl | 3.82 | 188 | 146 | 120 | 15 | 104 | 121 | 24 |
| Atrazine-desisopropyl | 2.62 | 174 | 132 | 120 | 15 | 96 | 120 | 15 |
| Azinphos-ethyl | 12.9 | 346 | 137 | 80 | 20 | 97 | 80 | 32 |
| Azinphos-methyl | 10.03 | 318 | 132 | 80 | 8 | 125 | 80 | 12 |
| Azoxystrobin | 10.113 | 404.02 | 372 | 95 | 12 | 344 | 95 | 20 |
| Bifenthrin | 18.38 | 440.2 | 181.1 | 94 | 6 | 166 | 94 | 46 |
| Buprofezin | 16.83 | 306 | 201 | 120 | 10 | 116 | 120 | 15 |
| Carbendazim | 3.91 | 192 | 160 | 95 | 17 | 132 | 95 | 25 |
| Carbofuran | 6.53 | 222 | 165 | 120 | 10 | 123 | 120 | 15 |
| Carbofuran-3-hydroxy | 2.75 | 255 | 220 | 70 | 5 | 163 | 70 | 15 |
| Chlorfenvinphos | 14.53 | 359 | 155 | 120 | 10 | 127 | 120 | 15 |
| Chlorpyrifos | 17.02 | 350 | 198 | 92 | 13 | 97 | 92 | 33 |
| Chlothianidin | 2.33 | 250 | 169 | 86 | 9 | 132 | 89 | 5 |
| Coumaphos | 15.4 | 363 | 335 | 134 | 10 | 307 | 134 | 10 |
| Cyhalotrhin | 18.1 | 467.1 | 225 | 66 | 10 | 141 | 66 | 46 |
| Diazinon | 14.57 | 305 | 169 | 128 | 21 | 153 | 128 | 17 |
| Dichlofenthion | 17.02 | 315 | 287 | 120 | 5 | 259 | 120 | 10 |
| Dimethoate | 3.06 | 230 | 199 | 80 | 5 | 171 | 80 | 10 |
| Diuron | 9.82 | 233 | 160 | 120 | 20 | 72 | 120 | 20 |
| DMA  DMF  DMPF | 2.92  5.88  2.88 | 122  150  163 | 107  132  122 | 111  111  111 | 18  10  15 | 77  107  107 | 111  111  111 | 42  15  15 |
| Ethion | 17.01 | 385 | 199 | 80 | 5 | 171 | 80 | 15 |
| Etofenprox | 18.23 | 394.2 | 359.2 | 66 | 10 | 177.1 | 66 | 10 |
| Fenitrothion | 12.45 | 278 | 125 | 140 | 15 | 109 | 121 | 12 |
| Fenoxon-Sulfone | 7.13 | 295 | 280 | 136 | 13 | 109 | 136 | 33 |
| Fenoxon-Sulfoxide | 14.33 | 279 | 247 | 114 | 5 | 169 | 114 | 13 |
| Fenthion | 14.33 | 279 | 247 | 114 | 5 | 169 | 114 | 13 |
| Fenthionoxon | 16.51 | 263 | 231 | 128 | 9 | 216 | 128 | 21 |
| Fenthion-Sulfone | 7.89 | 311 | 125 | 146 | 17 | 109 | 146 | 21 |
| Fenthion-Sulfoxide | 7.13 | 295 | 280 | 136 | 13 | 109 | 136 | 33 |
| Fluvalinate | 18.3 | 503 | 208 | 50 | 10 | 181 | 50 | 26 |
| Hexythiazox | 17.24 | 353 | 228 | 120 | 10 | 168 | 120 | 20 |
| Imazalil | 14.31 | 297 | 201 | 120 | 15 | 159 | 120 | 20 |
| Imidacloprid | 2.37 | 256 | 209 | 80 | 10 | 175 | 80 | 10 |
| Isoproturon | 9.45 | 207 | 165 | 120 | 10 | 72 | 120 | 20 |
| Malathion | 12.08 | 331 | 127 | 80 | 5 | 99 | 80 | 10 |
| Methiocarb | 11.45 | 226 | 169 | 80 | 5 | 121 | 80 | 10 |
| Methoalachlor | 13.01 | 284 | 252 | 120 | 10 | 176 | 120 | 15 |
| Molinate | 11.89 | 188 | 126 | 80 | 10 | 55 | 80 | 20 |
| Omethoate | 1.68 | 214 | 183 | 80 | 5 | 125 | 80 | 20 |
| Parathion-ethyl | 13.93 | 292 | 264 | 88 | 4 | 236 | 88 | 8 |
| Parathion-methyl | 10.77 | 264 | 232 | 110 | 5 | 125 | 120 | 20 |
| Prochloraz | 14.95 | 376 | 308 | 80 | 10 | 266 | 80 | 10 |
| Propanil | 11.48 | 218 | 162 | 120 | 15 | 127 | 120 | 20 |
| Propazine | 11.16 | 230 | 188 | 120 | 15 | 146 | 120 | 20 |
| Pyriproxifen | 17.01 | 322 | 227 | 120 | 10 | 185 | 120 | 10 |
| Simazine | 6.61 | 202 | 132 | 120 | 20 | 124 | 120 | 20 |
| Spinosyn A | 16.85 | 732.5 | 142.1 | 190 | 25 | 98.1 | 190 | 65 |
| Spinosyn D | 17.41 | 746.5 | 142.1 | 190 | 25 | 98.1 | 190 | 69 |
| Tebuconazole | 14.31 | 308 | 125 | 95 | 25 | 70 | 95 | 21 |
| Terbumeton | 11.46 | 226 | 170 | 95 | 17 | 114 | 95 | 25 |
| Terbumeton-desethyl | 7.2 | 198 | 142 | 90 | 13 | 86 | 90 | 25 |
| Terbuthylazine | 11.51 | 230 | 174 | 95 | 13 | 96 | 95 | 25 |
| Terbuthylazine-2-hydroxy | 7.5 | 212 | 156 | 95 | 13 | 86 | 95 | 25 |
| Terbuthylazine-deethyl | 7.51 | 202 | 146 | 95 | 13 | 79 | 95 | 25 |
| Terbutryn | 13.22 | 242 | 186 | 120 | 15 | 71 | 120 | 20 |
| Thiabendazole | 5.3 | 202 | 175 | 95 | 25 | 131 | 95 | 25 |
| Thiametoxam | 3.09 | 292 | 211 | 78 | 10 | 132 | 78 | 10 |
| Tolclofos-methyl | 15.03 | 301 | 269 | 120 | 15 | 125 | 115 | 12 |

# **Table S5**. Analytical conditions used for the analysis of pharmaceuticals.

| **Compound** | **Adduct/charge** | **Precursor Mass (m/z)** | **Confirming Peak** **(m/z)** | **RT (min)** | **R^2^** | **IS** |
| --- | --- | --- | --- | --- | --- | --- |
| 1H Benzotriazole | [M+H]^+^ | 120.0556 | 65.0386 | 5.0 | 0.9963 | BenzotriazoleD4 |
| 4-Hydroxidiclofenac | [M+H]^-^ | 310.0043 | 266.0144 | 10.0 | 0.9981 | Valsartan D3 |
| 5-methyl-1-H-Benzotriazole | [M+H]^+^ | 134.0713 | 79.0542 | 7.3 | 0.9961 | BenzotriazoleD5 |
| Acetaminophen | [M+H]^+^ | 152.0706 | 110.0600 | 3.0 | 0.9982 | AcetaminophenD4 |
| Acridone | [M+H]^+^ | 196.0757 | 167.0721 | 10.5 | 0.9968 | CarbamazepineD10 |
| Alprazolam | [M+H]^+^ | 309.0901 | 281.0713 | 12.7 | 0.9961 | AlprazolamD5 |
| Amantadine | [M+H]^+^ | 152.1434 | 135.1166 | 4.7 | 0.9587 | TrimethoprimD9 |
| Atenolol | [M+H]^+^ | 267.1703 | 190.0863 | 2.7 | 0.9994 | AtenololD7 |
| Atorvastatin | [M+H]^-^ | 557.2457 | 278.1350 | 13.1 | 0.9994 | Pentobarbital D5 |
| Benzoylecgonine | [M+H]^+^ | 290.1387 | 168.1020 | 5.9 | 0.9902 | SulfamethazineD4 |
| Bezafibrate | [M+H]^+^ | 362.1154 | 316.1099 | 13.5 | 0.9988 | BezafibratreD4 |
| Caffeine | [M+H]^+^ | 195.0877 | 138.0663 | 4.5 | 0.9990 | Caffeine13C3 |
| Carbamazepine | [M+H]^+^ | 237.1022 | 194.0967 | 11.9 | 0.9954 | CarbamazepineD10 |
| CBZ-10,11-epoxide | [M+H]^+^ | 253.0972 | 180.0810 | 9.7 | 0.9990 | CarbamazepineD10 |
| Chloramphenicol | [M+H]^-^ | 321.0051 | 152.0353 | 8.9 | 0.9966 | Pentobarbital D5 |
| Ciprofloxacin | [M+H]^+^ | 332.1405 | 288.1506 | 5.6 | 0.9967 | CiprofloxacinD8 |
| Citalopram | [M+H]^+^ | 325.1711 | 109.0448 | 10.5 | 0.9928 | CarbamazepineD10 |
| Clarithromycin | [M+H]^+^ | 748.4841 | 158.1175 | 12.4 | 0.9979 | CarbamazepineD10 |
| Coca-ethylene | [M+H]^+^ | 318.1700 | 196.1335 | 8.6 | 0.9993 | CocaineD5 |
| Cocaine | [M+H]^+^ | 304.1543 | 182.1178 | 7.2 | 0.9986 | CocaineD5 |
| Codeine | [M+H]^+^ | 300.1594 | 165.0699 | 3.5 | 0.9990 | CodeineD3 |
| Cotinine | [M+H]^+^ | 177.1022 | 80.0496 | 1.7 | 0.9990 | CotinineD3 |
| Diazepam | [M+H]^+^ | 285.0789 | 154.0420 | 13.8 | 0.9955 | AtorvastatinD5 |
| Diclofenac | [M+H]^-^ | 294.0094 | 214.0425 | 12.2 | 0.9989 | Diclofenac 13C6 |
| Diltiazem | [M+H]^+^ | 415.1686 | 178.0321 | 11.0 | 0.9877 | CarbamazepineD10 |
| Erythromycin | [M+H]^+^ | 734.4685 | 158.1174 | 11.2 | 0.9985 | Erythromycin13CD3 |
| Fluconazole | [M+H]^+^ | 307.1113 | 220.0681 | 7.1 | 0.9993 | Fluconazole13C3 |
| Flufenamic_acid | [M+H]^+^ | 282.0742 | 264.0630 | 15.0 | 0.9988 | Diclofenac13C6 |
| Fluoxetine | [M+H]^+^ | 310.1413 | 265.1591 | 12.4 | 0.9971 | FluoxetineD5 |
| Furosemide | [M+H]^-^ | 329.0004 | 204.9845 | 7.6 | 0.9993 | Furosemide D5 |
| Hydrochlorothiazide | [M+H]^-^ | 295.9572 | 268.9455 | 4.1 | 0.9989 | Hydrochlorothiazide 13CD2 |
| Ibuprofen | [M+H]^-^ | 205.1234 | 159.1180 | 12.7 | 0.9954 | Ibuprofen D3 |
| Indomethacin | [M+H]^-^ | 356.0695 | 297.0554 | 12.4 | 0.9857 | Lorazepam D4 |
| Ketoprofen | [M+H]^+^ | 255.1016 | 105.0336 | 13.3 | 0.9979 | KetoprofenD3 |
| Lamotrigine | [M+H]^+^ | 256.0151 | 172.9667 | 6.0 | 0.9997 | Lamotrigine13CD3 |
| Lorazepam | [M+H]^+^ | 321.0192 | 275.0137 | 12.5 | 0.9980 | LorazepamD4 |
| Losartan | [M+H]^+^ | 423.1695 | 207.0919 | 13.0 | 0.9960 | IbersartanD6 |
| Mefenamic acid | [M+H]^-^ | 240.1030 | 196.1134 | 12.9 | 0.9931 | Pentobarbital D5 |
| Metformin | [M+H]^+^ | 130.1087 | 60.0560 | 1.3 | 0.9957 | MentforminD6 |
| Methadone | [M+H]^+^ | 310.2165 | 265.1587 | 12.4 | 0.9910 | CarbamazepineD10 |
| Metoprolol | [M+H]^+^ | 268.1907 | 116.1071 | 6.5 | 0.9997 | MetoprololD7 |
| N-acethyl SMX | [M+H]^+^ | 296.0700 | 134.0602 | 9.0 | 0.9973 | Sulfamethoxazole13C6 |
| Nalidixic acid | [M+H]^+^ | 233.0921 | 205.0610 | 11.4 | 0.9964 | VenlafaxineD6 |
| N-Desmethylcitalopram | [M+H]^+^ | 311.1560 | 109.0447 | 10.3 | 0.9933 | CarbamazepineD10 |
| Nicotine | [M+H]^+^ | 163.1235 | 117.0573 | 1.5 | 0.9968 | NicotineD4 |
| O-desmethylvenlafaxine | [M+H]^+^ | 264.1958 | 58.0655 | 5.7 | 0.9956 | VenlafaxineD6 |
| Omeprazole | [M+H]^+^ | 346.1220 | 198.0583 | 9.3 | 0.9990 | CarbamazepineD10 |
| Oseltamivir-CBX | [M+H]^+^ | 285.1809 | 138.0550 | 5.2 | 0.9918 | OseltamivirD3 |
| Oxazepam | [M+H]^+^ | 287.0582 | 241.0527 | 12.3 | 0.9994 | OxazepamD5 |
| Propyphenazone | [M+H]^+^ | 231.1492 | 189.1026 | 12.4 | 0.9996 | CarbamazepineD10 |
| Quetiapine | [M+H]^+^ | 384.1746 | 253.0794 | 10.0 | 0.9939 | Midazolam13C6 |
| Salbutamol | [M+H]^+^ | 240.1594 | 178.0756 | 2.5 | 0.9928 | CodeineD3 |
| Salicylic acid | [M+H]^-^ | 137.0244 | 93.0345 | 2.9 | 0.9994 | Sotalol D6 |
| Sitagliptin | [M+H]^+^ | 408.1254 | 174.0529 | 7.6 | 0.9990 | SitagliptinD4 |
| Sotalol | [M+H]^+^ | 273.1268 | 213.0694 | 2.6 | 0.9967 | SotalolD6 |
| Sulfamethazine | [M+H]^+^ | 279.0910 | 204.0440 | 6.0 | 0.9990 | SulfamethazineD4 |
| Sulfamethoxazole | [M+H]^+^ | 254.0594 | 156.0114 | 8.0 | 0.9987 | Sulfamethoxazole13C6 |
| Sulfapyridine | [M+H]^+^ | 250.0645 | 156.0115 | 4.8 | 0.9992 | CarbamazepineD10 |
| Temazepam | [M+H]^+^ | 301.0739 | 255.0684 | 13.1 | 0.9930 | OxazepamD5 |
| Tramadol | [M+H]^+^ | 264.1963 | 58.0654 | 6.5 | 0.9985 | Tramadol13CD3 |
| Triclocarban | [M+H]^-^ | 312.9708 | 159.9724 | 15.4 | 0.9994 | Triclosan 13C6 |
| Trimethoprim | [M+H]^+^ | 291.1452 | 230.1162 | 4.7 | 0.9980 | TrimethoprimD9 |
| Valsartan | [M+H]^+^ | 436.2343 | 207.0919 | 13.7 | 0.9993 | ValsartanD3 |
| Valsartan acid | [M+H]^-^ | 265.0731 | 165.0706 | 3.9 | 0.9988 | Valsartan acid D4 |
| Venlafaxine | [M+H]^+^ | 278.2115 | 58.0655 | 8.3 | 0.9984 | VenlafaxineD6 |
| Verapamil | [M+H]^+^ | 455.2904 | 165.0910 | 12.3 | 0.9971 | FluoxetineD5 |
| Warfarin | [M+H]^-^ | 307.0976 | 161.0244 | 11.3 | 0.9989 | Pentobarbital D5 |
| Zolpidem | [M+H]^+^ | 308.1758 | 235.1230 | 8.0 | 0.9912 | VenlafaxineD6 |

**Table S6**. Limit of detection (LOD), limit of quantification (LOQ) and percentage of recovery for the target compounds included in this study.

| **Compound type** | **Substance group** | **Name** | **LOD (ng L^-1^)** | **LOQ (ng L^-1^)** | **Recovery (%)** |
| --- | --- | --- | --- | --- | --- |
| **Pharmaceuticals** | Antibacterial antifungal | 1H Benzotriazole | 0.12 | 0.39 | 70 |
|  | Metabolite of diclofenac | 4-Hydroxidiclofenac | 0.97 | 3.24 | 62 |
|  | Metabolite of benzotriazole | 5-methyl-1H-benzotriazole | 0.26 | 0.88 | 91 |
|  | Analgesic | Acetaminophen | 0.12 | 0.41 | 66 |
|  | Anticancer activity | Acridone | 0.16 | 0.54 | 90 |
|  | Anxiolytic | Alprazolam | 0.42 | 1.40 | 103 |
|  | Parkinson's treatment | Amantadine | 0.13 | 0.43 | 97 |
|  | Cardiovascular system | Atenolol | 0.10 | 0.34 | 73 |
|  | Lipid regulator | Atorvastatin | 0.16 | 0.47 | 67 |
|  | Metabolit of cocaine | Benzoylecgonine | 0.32 | 1.08 | 110 |
|  | Lipid regulator | Bezafibrate | 0.14 | 0.48 | 108 |
|  | Psychoanaleptic | Caffeine | 0.14 | 0.46 | 14 |
|  | Antiepileptic | Carbamazepine | 0.11 | 0.35 | 108^a^ |
|  | Antiepileptic | Carbamazepine-10,11-epoxide | 0.19 | 0.64 | 92 |
|  | Antibacterial | Chloramphenicol | 0.86 | 2.85 | 111 |
|  | Antibacterial | Ciprofloxacin | 0.16 | 0.48 | 103 |
|  | Psychoanaleptic | Citalopram | 0.11 | 0.37 | 56 |
|  | Antibacterial | Clarithromycin | 0.15 | 0.50 | 65 |
|  | Metabolite of Cocaine | Coca-ethylene | 0.11 | 0.37 | 79 |
|  | CNS stimulant | Cocaine | 0.16 | 0.54 | 80 |
|  | Respiratory system drug | Codeine | 0.13 | 0.43 | 47 |
|  | Nervous system drug | Cotinine | 0.15 | 0.51 | 125 |
|  | Psycholeptic | Diazepam | 0.12 | 0.40 | 102 |
|  | Antiinflammatory | Diclofenac | 1.51 | 5.05 | 79 |
|  | Calcium Chanel blocker | Diltiazem | 0.14 | 0.47 | 60 |
|  | Antibiotic | Erythromycin | 0.27 | 0.82 | 28 |
|  | Antifungal | Fluconazole | 0.19 | 0.63 | 108 |
|  | Anti-inflammatory | Flufenamic acid | 1.67 | 5.00 | 83 |
|  | Psychoanaleptics | Fluoxetine | 0.10 | 0.31 | 16 |
|  | Diuretic | Furosemide | 0.18 | 0.60 | 72 |
|  | Diuretic | Hydrochlorothiazide | 0.04 | 0.15 | 112 |
|  | Anti-inflammatory | Ibuprofen | 0.14 | 0.48 | 181 |
|  | Anti-inflammatory | Indomethacin | 0.12 | 0.41 | 61 |
|  | Anti-inflammatory and antirheumatic | Ketoprofen | 0.36 | 1.18 | 109 |
|  | Antiepileptic | Lamotrigine | 0.14 | 0.46 | 96 |
|  | Anxiolytic | Lorazepam | 0.18 | 0.60 | 105 |
|  | Antihypertensive activity | Losartan | 0.23 | 0.77 | 37 |
|  | Non-steroidal anti-inflammatory drug (NSAID) | Mefenamic acid | 0.46 | 1.53 | 56 |
|  | Drugs used in diabetes | Metformin | 0.03 | 0.10 | 75 |
|  | Analgesics Anesthetics | Methadone | 0.02 | 0.07 | 58 |
|  | Cardiovascular system | Metoprolol | 0.06 | 0.20 | 63 |
|  | Metabolite of Sulfamethoxazole | N-acetyl sulfamethoxazole | 0.16 | 0.52 | 107 |
|  | Antibiotics Antibacterial | Nalidixic acid | 0.12 | 0.41 | 87 |
|  | Metabolite of Citalopram | N-Desmethylcitalopram | 0.08 | 0.25 | 89 |
|  |  | Nicotine | 0.03 | 0.10 | 55 |
|  | Antidepressant | O-Desmethylvenlafaxine | 0.19 | 0.63 | 70 |
|  | Proton-pump inhibitor | Omeprazole | 0.03 | 0.10 | 42 |
|  | Metabolite of oseltamivir | Oseltamivir carboxylate | 0.13 | 0.38 | 19 |
|  | Psycholeptics and anxiolytics | Oxazepam | 0.12 | 0.39 | 112 |
|  | Analgesic and antipyretic | Propyphenazone | 0.18 | 0.60 | 104 |
|  | Antipsychotic Agents | Quetiapine | 0.08 | 0.25 | 87 |
|  | Short-acting β2 adrenergic receptor agonist | Salbutamol | 0.17 | 0.50 | 43 |
|  | Non-steroidal anti-inflammatory drug (NSAID) | Salicylic acid | 0.75 | 2.50 | 129 |
|  | Drugs used in diabetes | Sitagliptin | 0.21 | 0.70 | 47 |
|  | Β-blocking agent | Sotalol | 0.07 | 0.22 | 85 |
|  | Antibiotic | Sulfamethazine | 0.32 | 1.06 | 70 |
|  | Anti-infective and antibacterial | Sulfamethoxazole | 0.24 | 0.80 | 75 |
|  | Veterinary Pharmaceutical | Sulfapyridine | 0.07 | 0.39 | 67 |
|  | Antidepressant, sedative, hypnotic and anticonvulsant | Temazepam | 0.33 | 1.00 | 115 |
|  | Analgesics/Anesthetics | Tramadol | 0.19 | 0.64 | 52 |
|  | Antibacterial | Triclocarban | 0.14 | 0.41 | 99 |
|  | Antibacterial | Trimethoprim | 0.14 | 0.47 | 96 |
|  | Cardiovascular system | Valsartan | 0.19 | 0.63 | 88 |
|  | Cardiovascular system | Valsartan acid | 0.61 | 2.05 | 81 |
|  | Psychoanalytic | Venlafaxine | 0.01 | 0.05 | 66 |
|  | Calcium channel blocking agent | Verapamil | 0.11 | 0.36 | 47 |
|  | Anticoagulant | Warfarin | 0.22 | 0.74 | 92 |
|  | Treatment of insomnia | Zolpidem | 0.03 | 0.10 | 109 |
| **Pesticides** | Neonicotinoid | Acetamiprid | 0.3 | 1 | 58 |
|  | Triazine | Atrazine | 1.3 | 1 | 58 |
|  | Triazine | Atrazine-Deethyl | 2 | 1 | 52 |
|  | Triazine | Atrazine-Deisopropyl | 2 | 1 | 51 |
|  | Strobilurin | Azoxystrobin | 1.5 | 4.5 | 72 |
|  | Benzimidazole | Carbendazim | 0.1 | 1 | 52 |
|  | Organophosphate | Diazinon | 0.2 | 1 | 78 |
|  | Organophosphate | Dimethoate | 1 | 1 | 53 |
|  | Phenylamine | Diuron | 1.3 | 3 | 51 |
|  | Amidine | DMPF | 1.3 | 10 | 70 |
|  | Organophosphate | Fenthion sulfoxide | 0.3 | 1 | 52 |
|  | Imidazole | Imazalil | 0.3 | 1 | 53 |
|  | Neonicotinoid | Imidacloprid | 0.8 | 1 | 57 |
|  | Imidazole | Prochloraz | 2 | 1 | 65 |
|  | Anilide | Propanil | 0.3 | 1 | 54 |
|  | Triazine | Simazine | 0.15 | 1 | 82 |
|  | Microorganism derived | Spinosad A | 0.015 | 1 | 72 |
|  | Microorganism derived | Spinosad D | 0.015 | 1 | 69 |
|  | Triazole | Tebuconazole | 0.15 | 1 | 80 |
|  | Triazine | Terbumeton | 0.015 | 1 | 75 |
|  | Triazine | Terbumeton deethyl | 0.015 | 1 | 72 |
|  | Triazine | Terbuthylazine | 0.015 | 1 | 70 |
|  | Triazine | Terbuthylazine-2OH | 0.15 | 1 | 69 |
|  | Triazine | Terbuthylazine-deethyl | 0.15 | 1 | 62 |
|  | Triazine | Terbutryn | 0.15 | 1 | 68 |
|  | Benzimidazole | Thiabendazole | 0.15 | 1 | 70 |

**Figure S1.** Comparison of chemical concentrations measured in grab samples and in the POCIS samples on D0 and D14. Observations outside dotted lines represent deviations above one order of magnitude between both methods. ρ is the Spearman´s rank correlation coefficient between the concentrations obtained with the grab and the POCIS sampling methods. The asterisks indicate a Spearman´s rank p-value of ≤ 0.05 (*); ≤ 0.01 (**); and ≤ 0.001 (***).

**
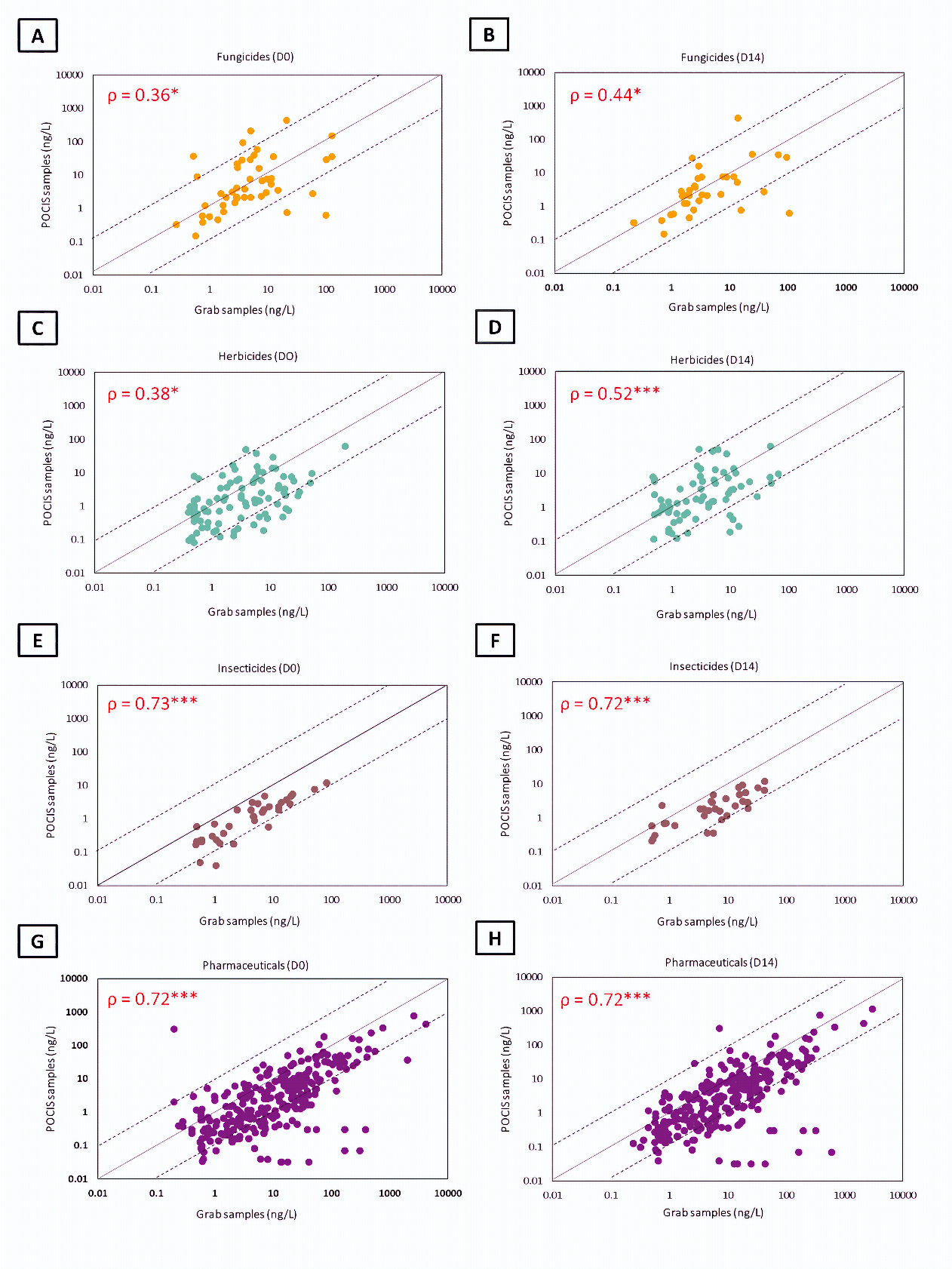
**

**Figure S2.** Mean elimination rate by the constructed wetland for each group of compounds.


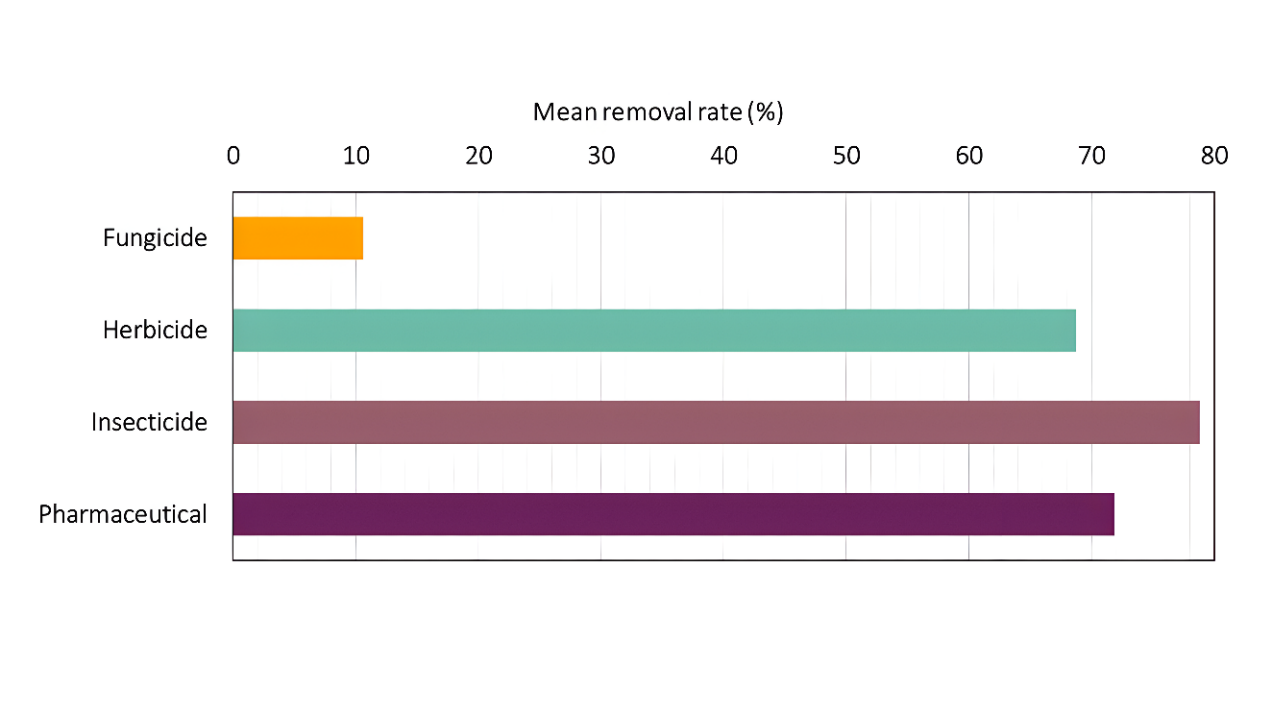

Supplement: Supplementary file 1 — Supplementary file1 (DOCX 2301 KB) [file 11356_2024_31968_MOESM1_ESM.docx]
